# Dual‐Interphase‐Stabilizing Sulfolane‐Based Electrolytes for High‐Voltage and High‐Safety Lithium Metal Batteries

**DOI:** 10.1002/advs.202410129

**Published:** 2024-10-01

**Authors:** Junhua Zhou, Chi Zhang, Huimin Wang, Yanpeng Guo, Chuan Xie, Yufeng Luo, Chao Wang, Shujing Wen, Jiehua Cai, Wancheng Yu, Fan Chen, Yufei Zhang, Qiyao Huang, Zijian Zheng

**Affiliations:** ^1^ School of Fashion and Textiles The Hong Kong Polytechnic University Hong Kong SAR 999077 China; ^2^ Department of Applied Biology and Chemical Technology The Hong Kong Polytechnic University Hong Kong SAR 999077 China; ^3^ Research Institute for Intelligent Wearable Systems The Hong Kong Polytechnic University Hong Kong SAR 999077 China; ^4^ Research Institute for Smart Energy The Hong Kong Polytechnic University Hong Kong SAR 999077 China

**Keywords:** high‐voltage electrolytes, interphase, lithium metal batteries (LMBs), safety, sulfolane

## Abstract

High‐voltage Li metal battery (HV‐LMB) is one of the most promising energy storage technologies to achieve ultrahigh energy density. Nevertheless, electrolytes reported to date are difficult to simultaneously stabilize the Li metal anode and high‐voltage cathode, especially without the assistance of expensive and corrosive high‐concentration Li salts. Herein, a dual‐interphase‐stabilizing (DIS) and safe electrolyte that bypasses the high‐concentration Li salt is reported. The electrolyte consists of high‐flash‐point sulfolane as solvent, molecular‐orbital‐engineered additives that enable stable B‐F rich cathodic interphase, and unique C‐F rich organic anodic interphase. The stable cycling of both Li metal anode and 4.75 V‐LiCoO_2_ cathode in the DIS electrolyte (> 500 cycles) is demonstrated. HV‐LMB pouch cells of a high energy density (435 Wh kg^−1^) can sustainably operate for more than 100 cycles. Moreover, the low cost and high thermal stability of the DIS electrolyte offer superior cost‐effectiveness and safety for large‐scale applications of HV‐LMBs in the future.

## Graphical abstract

A safe and stable sulfolane‐based electrolyte is designed by molecular orbital engineering, which promotes the formation of robust solid electrolyte interphase that enables the stable operation of Li metal anode and high‐voltage cathode. This work demonstrates the effectiveness of regulating electrolyte chemistry, offering great potential for large‐scale applications of high‐voltage lithium metal batteries.

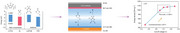

## 1 Introduction

**Figure 1:**
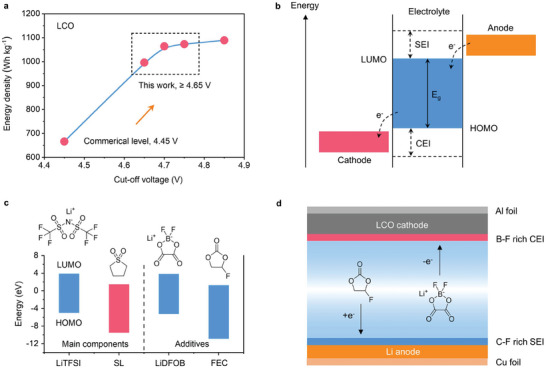
Advantages of high‐voltage LCO cathode and design principle of this work. a) Theoretical energy density of LCO cathode as a function of cut‐off voltage. b) Energy diagram of electrolyte. Four key factors influence the electrochemical window of electrolyte, including the HOMO, LUMO, SEI at the anode side, and that at the cathode side (CEI). c) Comparison of the HOMO and LUMO of Li salt (LiTFSI), solvent (SL), and additives (LiDFOB and FEC) used in this work. d) The functional mechanism of FEC and LiDFOB additives.

Enhancing the energy density of batteries represents a pivotal objective of battery development as it directly correlates with extended driving ranges for electric vehicles, the miniaturization of consumer electronics, and the reduction of costs associated with electrical grid storage solutions.^[^ ^1^, ^2^, ^3^ ^]^ High‐voltage lithium metal battery (HV‐LMB) stands as one of the most auspicious contenders to achieve high energy density, due to the use of lithium metal as the anode and high‐voltage material as the cathode.^[^ ^4^, ^5^, ^6^, ^7^, ^8^ ^]^ E.g., the maximum voltage of commercially available lithium cobalt oxide (LCO) is 4.45 V, which confers an energy density of ≈666 Wh kg^−1^ based on the LCO electrode material (**Figure** **1**a; Figure S1 and Table S1, Supporting Information). Advancing the voltage to 4.65 V, and further to 4.7 V or 4.75 V, would elevate the energy density to 996 Wh kg^−1^, 1064 Wh kg^−1^, and 1073 Wh kg^−1^, respectively.

However, the development of HV‐LMBs is significantly hampered by the lack of suitable electrolytes that can simultaneously stabilize both the anode/electrolyte interface and the cathode/electrolyte interface. Ether‐based electrolytes offer a stable solid electrolyte interphase (SEI) at the anode, and markedly improve the Li plating/stripping efficiency. However, they suffer from poor oxidation stability (< 4 V vs Li/ Li^+^), which impedes the use of high‐voltage cathode materials.^[^ ^9^, ^10^, ^11^ ^]^ Carbonate ester‐based electrolytes, on the other hand, can form stable cathode electrolyte interphase (CEI) and show good high‐voltage stability (> 4.5 V vs Li/ Li^+^), but their Coulombic efficiency (CE) toward Li metal anode is very poor.^[^ ^12^, ^13^, ^14^, ^15^, ^16^ ^]^ The problem has been partially addressed by using high‐concentration electrolytes (HCEs), which improve the Li plating/stripping efficiency and oxidation stability by modulating the solvation structure within the electrolyte. However, there are several disadvantages of using HCEs, including 1) increased viscosity that deteriorates Li^+^ conductivity and battery's rate performance,^[^ ^17^ ^]^ 2) high cost of the substantial quantity of specialized lithium salts, such as lithium bis(trifluoromethanesulfonyl)imide (LiTFSI), posing significant economic challenges for large‐scale application,^[^ ^18^ ^]^ and 3) severe corrosion of aluminum foil, the current collector of cathode, at elevated voltages (≈4 V vs Li^+^/Li) as a result of the presence of iminium ions in LiTFSI.^[^ ^19^ ^]^

Sulfolane (SL), an emerging electrolyte solvent, exhibits considerable promise for HV‐LMBs. SL is featured by its exceptional oxidation stability exceeding 5 V (vs Li^+^/Li), because SL's energy level of the highest occupied molecular orbital (HOMO) is very low.^[^ ^20^ ^]^ Additionally, SL's high flash point (166 °C) will significantly enhance the safety profile of the battery,^[^ ^21^ ^]^ considering that commercial ester‐ and ether‐based electrolytes are highly flammable. As a solvent commonly utilized in the chemical industry, SL also offers a cost advantage.^[^ ^22^ ^]^ However, the energy level of the lowest unoccupied molecular orbital (LUMO) of SL is too low to be compatible with the Li metal anode. Although the HCE approach can improve SL's compatibility to Li metal anode,^[^ ^21^, ^23^, ^24^ ^]^ it is not desirable in the industry due to the above‐mentioned drawbacks.

Herein, we report a dual‐interphase‐stabilizing (DIS), SL‐based electrolyte for high‐safety HV‐LMBs. We bypass the high‐concentration strategy and instead incorporate fluoroethylene carbonate (FEC) and lithium difluoro(oxalato)borate (LiDFOB) as additives to the electrolyte. This DIS electrolyte enables the formation of a unique organic fluorine (C‐F) rich SEI, which effectively prevents the decomposition of SL at the anode, as well as the formation of a B‐F rich CEI at the cathode to allow high‐voltage operation. As a result, the LMB cell simultaneously acquires stable cycling of Li metal anode for over 500 cycles with a high average CE of 98.8%, and stable operation of LCO cathode up to 4.75 V (Figure 1a). Consequently, our assembled HV‐LMB pouch cell exhibits a high energy density of 435 Wh kg^−1^ and stable cycling behavior for over 100 cycles with a high‐capacity retention of 83.2%, together with enhanced thermal stability.

## 2 Results and Discussions

### 2.1 Design Principle and Components of Electrolytes

Of note, the absolute value of the energy gap between LUMO and HOMO cannot be used to calculate the electrochemical window of electrolytes.^[^ ^25^ ^]^ However, the relative value of LUMO and HOMO can predict the stability of compounds at low and high voltage respectively in the corresponding electrolyte system.^[^ ^19^ ^]^ In this work, the LUMO and HOMO energy levels were calculated to screen solvents and additives (Figure 1b). To achieve an electrolyte with high oxidation stability, the SL with extremely low HOMO (−9.5 eV; Figure 1c; Figure S2 and Table S2, Supporting Information) is chosen as the solvent. However, the low LUMO (1.4 eV) of SL implies that it will be reduced easily under low potential. FEC possessing lower LUMO (1.2 eV) will be reduced preferentially to generate an organic‐F‐rich (C‐F) SEI layer on the anode surface, which is stable enough to prevent the continuous decomposition of SL solvent (Figure 1d; Figure S3, Supporting Information). On the other hand, LiDFOB of high HOMO (−5.2 eV) will be oxidized to form the stable B‐F rich CEI. The LiTFSI as the lithium salt also benefits to forming the stable SEI and CEI, and its side effect about causing the Al corrosion can be suppressed by applying the FEC and LiDFOB additives. As a result, the DIS electrolyte can enable the sustainable operation of HV‐LMBs.

As a proof of concept, we formulated three distinct electrolytes with different amounts of additives, being 1) 2 M LiTFSI in SL (LS), 2) 2 M LiTFSI in SL with 10 vol.% FEC (LS‐FEC), and 3) 2 M LiTFSI and 0.2 M LiDFOB in SL with 10 vol.% FEC (LS‐DIS). All electrolytes show high oxidation potential (> 5.3 V), good wettability toward commercial separators (contact angle < 80°), good Li^+^ conductivity at room temperature (1–3 mS cm^−1^), low activation energy (13–22 kJ mol^−1^), and good Li^+^ transference number (≈0.4; Figures S4–7 and Table S3, Supporting Information). The identical peak positions of LS, LS‐FEC, and LS‐DIS in Raman spectra (Figure S8, Supporting Information) indicate that the tiny amounts of additives do not alter the solvation structure obviously. Moreover, the disappeared plastic crystal temperature (T_pc_) and melting temperature (T_m_) indicate the prolonged liquid range of SL after introducing Li salt and additives (Figure S9, Supporting Information). The use of SL as a solvent also brings a price advantage to the electrolyte (SL, 337.1 US$/kg; common carbonate esters, 2136.78 US$/kg; Table S4, Supporting Information).

### 2.2 Efficiency and Morphology Evolution of Li Metal Anode

**Figure 2:**
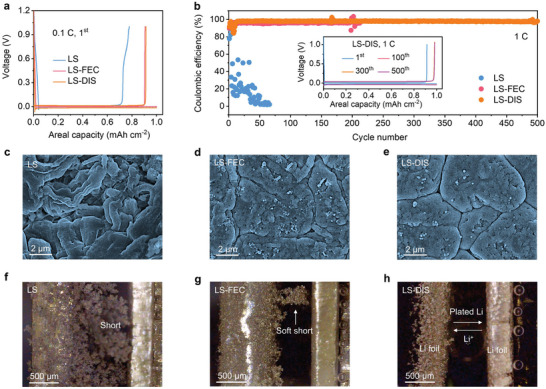
Electrochemical performance and morphology evolution of Li metal anode in various electrolytes. a) Charge‐discharge curves in the 1^st^ cycle at 0.1 C (1 C = 1 mA cm^−2^). b) CE plots during 500 cycles at 1 C. The insets in (b) show overlapped voltage profiles of the Li metal anode in the LS‐DIS during cycling. c–e) SEM images of Li metal after 50 cycles. f–h) Operando optical microscopy images showing the cross section of symmetric Li||Li cells after Li plating at 1 C for 1 h. The label in (h) shows the direction of Li^+^ flux and Li metal deposition.

Li||Cu asymmetrical cells (capacity limitation, 1 mAh cm^−2^) were tested to evaluate the Li metal plating/stripping efficiency in various electrolytes. LS shows an extremely low initial CE of 77.8% at 0.1 C (**Figure** **2**a, 1C = 1 mA cm^−2^) and cannot cycle at 1 C (Figure 2b). In contrast, LS‐FEC exhibits greatly enhanced initial CE of 90.7% and can stably operate for ≈200 cycles with an average CE of 97.5%. The findings, in conjunction with the cyclic voltammetry (CV) curves presented in Figure S10 (Supporting Information), indicate that the SL is incompatible with Li metal, and the compatibility can be partially mitigated by introducing the FEC additive. To assess how FEC influences the efficiency of Li plating/stripping during long‐term cycling, Li||Cu cells were tested in the LS‐DIS with various ratios of FEC. The cell in LS‐DIS with 10% FEC shows high and stable CE for more than 500 cycles (Figure S14, Supporting Information). These results exhibited the long‐term stability of our electrolytes during the charge/discharge cycles, with no apparent degradation of their positive impact on the lithium inventory.^[^ ^26^, ^27^ ^]^ Importantly, the cycling sustainability of Li metal is substantially enhanced to over 500 cycles with an average CE of 98.8% (Figure 2b) in the LS‐DIS, indicating that the LiDFOB additives further improve the compatibility of SL toward Li metal. The enhanced performance of the LS‐DIS electrolyte is also corroborated by the stable operation of Li||Cu and Li||Li cells across a range of current densities from 0.1 C to 10 C (Figures S11 and S12, Supporting Information).

The various morphological evolutions over cycling explain the distinct CE difference in each electrolyte. In LS, the surface of the Li metal anode shows a porous structure with moss‐like dendrites extending for tens of microns after 50 cycles (Figure 2c; Figure S15A, Supporting Information). In LS‐FEC, the Li metal surface is much denser, showing Li chunks of a few microns in diameter and a tiny amount of dendrites (Figure 2d; Figure S15b, Supporting Information). The Li metal plated in LS‐DIS exhibits the densest structure and dendrite‐free characteristic (Figure 2e; Figure S15c, Supporting Information). The reduced specific surface area implies less decomposition of the electrolyte upon the Li metal, thus leading to higher plating/stripping efficiency.^[^ ^28^ ^]^ Operando microscopy reveals the Li||Li symmetric cell running in LS shorts after only 0.5 h of plating at a 1 C rate, accompanied by a sudden increase in potential (Figure 2f; Figure S16a and Video S1, Supporting Information). In LS‐FEC, much fewer Li dendrites form, although a soft short is observed at the end of plating (Figure 2g; Figure S16b and Video S2, Supporting Information). In contrast, no short is observed when running the cell in LS‐DIS (Figure 2h; Figure S16c and Video S3, Supporting Information). These results align well with the electrochemical data discussed above.

### 2.3 Interphase between Electrolyte and Li Metal Anode

**Figure 3:**
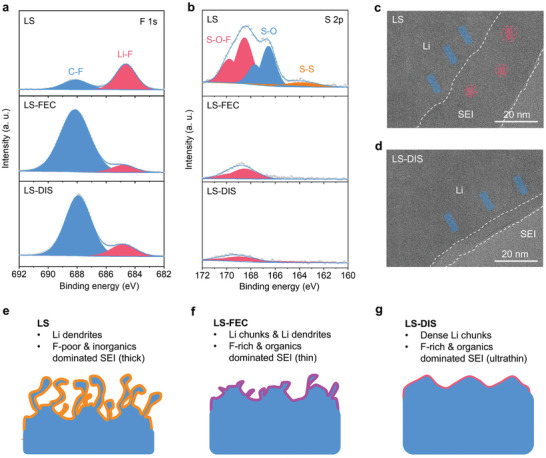
Composition and microstructure of SEI formed in various electrolytes. a,b) F 1s and S 2p XPS patterns of Li metal after 50 cycles. c,d) Cryo‐TEM images of lithium metal deposited on Cu grids. e–g) Schematics showing the bulk and interphase structure of cycled Li metal.

The composition of SEI produced in different electrolytes was examined using F 1s X‐ray photoelectron spectroscopy (XPS). As depicted in **Figure** **3**a, two principal peaks at ≈688.2 eV and 684.6 eV are attributed to organic C‐F and inorganic Li‐F species, respectively.^[^ ^6^ ^]^ The SEI formed in LS (Figure 3a, the top panel) possesses a slight abundance of inorganic F species over organic F compounds, which is a typical characteristic of the decomposition of LiTFSI. In contrast, organic F compounds dominate the SEIs formed in LS‐FEC and LS‐DIS (Figure 3b, the middle and bottom panels, and Figure S17a, Supporting Information), which can be ascribed to the decomposition of FEC.^[^ ^29^, ^30^ ^]^ The overall ratio of F species in the SEI is largely increased (Figure S17b, Supporting Information). Moreover, after the addition of the LiDFOB additive, the SEI in LS‐DIS displays slightly larger amount of inorganic Li‐F compounds compared with the LS‐FEC. The XPS depth profiles of F 1s also support the discussions (Figure S18, Supporting Information).

The SEI characteristic plays a pivotal role in the stability of electrolytes. In S 2p XPS spectra of LS (Figure 3b, the top panel), three deconvoluted peaks at ≈168.4, 166.5, and 163.9 eV can be indexed to the S‐O‐F, S‐O, and S‐S species, respectively.^[^ ^21^ ^]^ These species mainly are byproducts of the decomposition of SL solvent, considering the low LUMO of SL in the electrolytes (1.4 eV, Figure 1c). In LS‐FEC and LS‐DIS, the amounts of S species are greatly reduced (Figure 3b, the middle and bottom panels, and Figure S17, Supporting Information). This result suggests that the organic‐F rich SEIs can suppress the decomposition of SL, leading to enhanced cycling stability of Li metal. The composition of SEI and stability of electrolytes are also revealed by C 1s, O 1s, B 1s, and N 1s spectra (Figure S19, Supporting Information).

Cryogenic transmission electron microscopy (Cryo‐TEM) provides further insights into the microstructure of the SEI formed in various electrolytes. The SEI typically appears as a layer with slightly lower contrast on the lithium metal surface.^[^ ^31^ ^]^ Based on the structure observed in Figure 3c,d and Figure S20 (Supporting Information), the thickness of SEI is determined to be ≈36, 15, and 6 nm in the LS, LS‐FEC, and LS‐DIS, respectively. A thinner SEI is generally associated with increased stability, as also corroborated by EIS results shown in Figure S21 (Supporting Information), where LS exhibited a much higher interphase resistance in comparison to LS‐FEC and LS‐DIS. Moreover, the SEI in LS (Figure 3c) contains small crystal grains (≈4 nm, inorganic compounds) randomly dispersed in an amorphous matrix (organic species). In contrast, no similar crystalline domain is found in the LS‐FEC and LS‐DIS, indicating the dominance of organic components within their SEI.

In summary of the microscopic and spectroscopic studies, mossy Li dendrites with thick SEI are formed in LS during cycling (Figure 3e). The unstable SEI contains a small proportion of F species, which is dominated by inorganic compounds. In comparison, the addition of FEC in LS‐FEC and LS‐DIS generates organic‐F rich SEI, and promotes the formation of much denser Li chunks with a thin SEI layer (Figure 3f,g). The organic F‐rich SEI is steady enough to inhibit the continuous decomposition of SL. Notably, this is different from the conclusions in most reported works, where inorganic LiF‐rich SEI formed in HCEs are often preferred for achieving high Li plating/stripping efficiency.^[^ ^6^, ^32^ ^]^ This means that our work recommends a new interphase chemistry, that the additive‐assisted organic‐rich SEI can also enhance the compatibility of low‐LUMO solvent with Li metal. To further validate this hypothesis, an electrolyte based on succinonitrile (SN) with a similarly low LUMO (−1.1 eV)^[^ ^33^ ^]^ was also investigated. Our results (Figure S22, Supporting Information) demonstrate that the use of FEC additive remains essential. Besides, the LiDFOB additive can further optimize the morphology of Li metal and SEI (Figure 3g), rendering the cycling of Li anode outstanding in LS‐DIS.

### 2.4 High‐Voltage Compatibility of Electrolyte

**Figure 4:**
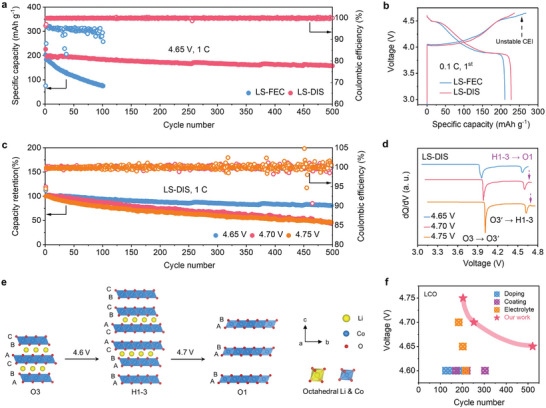
Electrochemical performance of high‐voltage LCO cathode. a,b) Cycling behavior during 500 cycles at 1 C and charge‐discharge curves in the 1^st^ cycle at 0.1 C (1 C = 220 mA g^−1^) with the voltage range of 3–4.65 V in the LS‐FEC and LS‐DIS. c,d) Cycling behavior during 500 cycles at 1 C and differential plots derived from charge‐discharge curves in the 1^st^ cycle at 0.1 C with the voltage range of 3–4.65, 3–4.70, and 3–4.75 V in the LS‐DIS. e) Structure evolution of LCO during the charging process. The O represents octahedral sites and the number is the stacking sequence of oxygen layers. Accordingly, the ABCABC, ABABCACABCBC, and ABABAB stacking orders can be assigned to the O3, H1‐3 (hybridized O1‐O3), and O1 structures respectively. f) Comparison of cycling stability of high‐voltage LCO (≥ 4.6 V) with doping, coating, and electrolyte modification strategies (Table S5, Supporting Information). The cycle number in (f) represents the value when the battery's capacity fades to 80%.

The electrochemical behavior of commercial LCO cathode (loading: 2.4 mAh cm^−2^) was first evaluated at 4.65 V in various SL‐based electrolytes. In LS, the LCO experiences a rapid failure during the first charge at ≈4.3 V as a result of the corrosion of the Al foil by LiTFSI (Figure S24, Supporting Information).^[^ ^34^ ^]^ After introducing the FEC additive, the corrosion is suppressed in LS‐FEC. However, the poor average CE of 93.80% during 100 cycles in LS‐FEC indicates the lack of stable CEI, which leads to increased voltage polarization, and thereby severe capacity decay with retention of only 40.38% after 100 cycles (**Figure** **4**a; Figure S25, Supporting Information). In contrast, when operating in LS‐DIS, the LCO cathode delivers high reversible specific capacity (228 mAh g^−1^) and CE (96.38%) in the initial cycle at 0.1 C (1 C = 220 mA g^−1^, Figure 4b). More importantly, the LCO cathode displays remarkably stable cycling capabilities at 1 C. After 500 cycles, the capacity retention and average CE are 80.8% and 99.93%, respectively (Figure 4a). The performance is superior to all reported works that use doping,^[^ ^35^, ^36^, ^37^ ^]^ coating,^[^ ^38^, ^39^, ^40^ ^]^ and electrolyte^[^ ^41^, ^42^, ^43^ ^]^ modification to optimize high‐voltage LCO cathode (Figure 4f; Table S5, Supporting Information). The LCO in LS‐DIS also exhibits desirable fast‐charging performance, with a specific capacity of 98 mAh g^−1^ even at 5 C (Figure S26, Supporting Information). These results mean that suppressing the corrosion of Al foil and stabilizing CEI are two key points regarding electrolyte design for high‐voltage cathode.

Importantly, LS‐DIS allows for smooth running of LCO at very high voltages up to 4.75 V. LCO in LS‐DIS shows high reversible capacity at 0.1 C in the initial cycle (4.65 V: 228 mAh g^−1^; 4.7 V: 240 mAh g^−1^; 4.75 V: 243 mAh g^−1^; Figure S27, Supporting Information). The capacity gain from 4.7 to 4.75 V is subtle, suggesting that there are diminishing returns in pushing the upper cut‐off voltage beyond 4.7 V for the LCO cathode. The similar initial CE of LCO at various voltages (4.65 V: 94.01%; 4.7 V: 94.94%; 4.75 V: 94.45%) reveals the robust oxidation stability of LS‐DIS. Moreover, the capacity retention of LCO at 4.7 V and 4.75 V after 500 cycles at 1 C are 50.12% and 49.65% respectively (Figure 4c). Although the values are much lower than that at 4.65 V (80.8%), they still significantly surpass all reported results for high‐voltage LCO systems (Figure 4f; Table S5, Supporting Information). Notably, the capacity decay at 4.7 and 4.75 V originates from the structural deterioration of LCO rather than the LS‐DIS electrolyte; based on differential plots derived from charge‐discharge curves (Figure 4d), an extra peak is found ≈4.7 V, attributed to the irreversible structural transition of LCO from H1‐3 to O1 (Figure 4e).^[^ ^37^ ^]^ In addition, the stability of LS‐DIS in Li‐LCO cells was also tested at high temperature (50 °C), in view of the possibly increased interphase reactions and transition metal dissolution.^[^ ^44^ ^]^ After 100 cycles, the capacity retention and average CE of 4.65 V‐LCO are 91.53% and 99.92%, respectively (Figure S35, Supporting Information). Such a high CE showcases the stability of our electrolyte at elevated temperature. The effectiveness of the LS‐DIS is also proved using a commercial NCM811 cathode, which shows desirable stability with capacity retention of 81.09% and average CE of 99.92% after 500 cycles at 1 C at 4.7 V (Figure S28, Supporting Information).

### 2.5 Interphase and Bulk Structure of Cycled LCO Cathode

**Figure 5:**
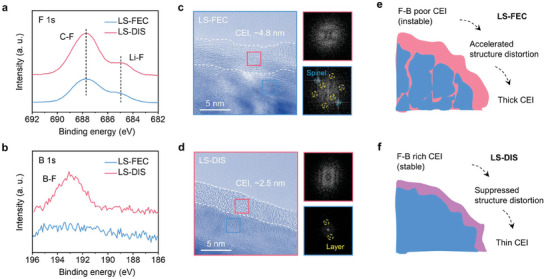
Failure analyses of high‐voltage LCO cathode in various electrolytes. a,b) F 1s and B 1s XPS patterns of LCO cathode after 100 cycles. c,d) TEM images and corresponding fast Fourier transform patterns showing the interphase and bulk structure of cycled LCO. e,f) Schematics delivering the failure mechanism of high‐voltage LCO.

The LS‐DIS generates highly stable CEI. The LiDFOB additive in LS‐DIS with high HOMO (−5.2 eV, Figure 1c), will be oxidized at high potential preferentially. Accordingly, the generated F‐ and B‐containing species can improve the stability of CEI (**Figure** **5**a,b; Figure S29, Supporting Information).^[^ ^45^, ^46^ ^]^ As a result, the CEI in LS‐DIS is thinner than that in LS‐FEC (LS‐FEC, 4.8 nm; LS‐DIS, 2.5 nm, Figure 5c,d), which leads to the decreased interface impedance (LS‐FEC, 69 Ω; LS‐DIS, 38 Ω; Figure S30, Supporting Information).

The CEI in LS‐FEC exhibits reduced stability, leading to the structure distortion of the LCO after being continuously attacked by electrolytes. The cycled LCO in LS‐DIS maintains its layered structure, whereas was partially transferred to the spinel structure in LS‐FEC (Figure 5c,d). From XRD results, the (003) diffraction peak of the cycled LCO in LS‐FEC shifts toward a lower angle, signifying an increase in the interlayer space along the c‐axis, and thus large volume expansion (Figure S31, Supporting Information). The stable CEI formed in LS‐DIS also eliminates the erosion of Al foil and reduces leakage current at high voltage (Figure S32, Supporting Information).

Overall, the unstable CEI formed in LS‐FEC at high voltage causes electrolyte depletion and crack formation in LCO (Figure S31, Supporting Information), and the exposed surface of LCO, in turn, leads to thick CEI and battery failure (Figure 5e). On the contrary, the LiDFOB in LS‐DIS can facilitate the generation of B‐ and F‐rich CEI, which curtails electrolyte decomposition and crack propagation, thereby ensuring exceptional cycling performance of the LCO cathode at high voltages (Figure 5f).

### 2.6 HV‐LMB Pouch Cell and Safety Evaluation

**Figure 6:**
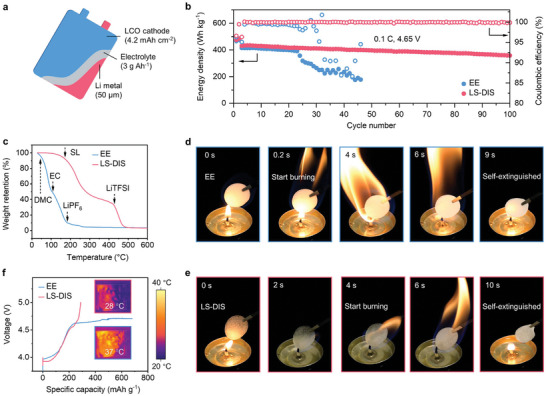
Pouch cell performance and safety evaluation. a) Schematic illustration of the Li||LCO pouch cell with a low N/P of 2.3. b) Energy densities of the pouch cell in the commercial ester‐based electrolyte (EE, 1 M LiPF_6_ in DMC: EC = 2:1 wt.%) and LS‐DIS operating at 3–4.65 V. c) TGA profiles of various electrolytes testing in N_2_. The arrows in (c) show the start point of weight loss for each component. d,e) Burning tests of glass fiber separators soaked with electrolytes. f) Over‐charge curves of LCO at 0.1 C. The insets exhibit the infrared thermal images at the final points of charging.

As a proof of concept of the practical potential of LS‐DIS, we prepared HV‐LMB pouch cells with a high‐loading cathode (LCO, 4.2 mAh cm^−2^), an ultrathin Li metal anode (50 µm), and lean electrolyte (3 g Ah^−1^), in which the capacity ratio of negative to positive electrode (N/P ratio) is ≈2.3 (**Figure** **6**a; Table S6, Supporting Information). The cell possesses high energy density (435 Wh kg^−1^) and can sustainably charge/discharge for over 100 cycles with a high‐capacity retention of 83.21% and an average CE of 99.95% at 0.1 C (Figure 6b). This performance is starkly contrasted with the rapid failure observed in similar pouch cells using a commercial ester‐based electrolyte (EE, 1 M LiPF_6_ in DMC: EC = 2:1 wt.%), which failed after just 20 cycles. The failure of pouch cells in the EE is attributed to the structure distortion of LCO and increased interphase impedance (Figure S33, Supporting Information).

Thermogravimetric analysis (TGA) reveals superior thermal stability of LS‐DIS. Due to the low flash point (17 °C) and high volatility of DMC, the EE exhibits significant weight loss once the temperature started to rise to 40 °C (Figure 6c; Figure S34a, Supporting Information). In stark contrast, SL has a much higher flash point (166 °C). Therefore, LS‐DIS demonstrated enhanced thermal resistance: weight loss is not observed until the temperature is raised to 170 °C.

Combustion tests further highlight the advantage of using SL‐based electrolyte against the commercial ester‐based electrolyte by applying glass fiber separators infiltrated with the LS‐DIS or EE. The EE‐imbibed separator ignites almost immediately (≈0.2 s), fueled by the low flash point of DMC leading to a vigorous flame and extinguishes only after the electrolyte is fully consumed (Figure 6d; Video S4, Supporting Information). Conversely, LS‐DIS resists ignition for 4 s due to its higher thermal stability and flash point (166 °C) (Figure 6e; Video S5, Supporting Information). After that, it also shows notably weaker flame and a much shorter self‐extinguishing time (SET) of 23 s per gram (s g^−1^) compared to 45 s g^−1^ for the EE (Figure S34b, Supporting Information).

The LS‐DIS ’s commendable stability significantly enhances the safety profile of high‐voltage LCO‐based pouch cells. Infrared thermal imagery (Figure 6f) shows that the LS‐DIS equipped pouch cell can be charged to 5 V and maintained a stable temperature of 28 °C. In contrast, cells using the EE struggle beyond 4.7 V exhibit a prolonged voltage plateau indicative of continuous electrolyte decomposition. The lability of EE at high voltage also causes obvious temperature rise to ≈37 °C and highlights potential safety risks upon overcharging.

## 3 Conclusions

In this study, we developed a novel DIS electrolyte that addresses the stability challenge of both cathodic and anodic interfaces of HV‐LMBs. The DIS electrolyte utilized high‐flash‐point SL solvent to enhance the thermal stability, and two molecular‐orbital‐engineered additives, FEC and LiDFOB, to stabilize the battery. We found that an organic C‐F rich SEI formed at the anode could effectively suppress the decomposition of SL and prevent dendrite formation, while decomposed LiDFOB formed a B‐F rich CEI and stabilize the cathode at high voltages. Using the DIS electrolyte, the Li metal anode and 4.75 V‐LCO cathode were cycled stability for more than 500 times. We demonstrated a high‐energy‐density (435 Wh kg^−1^) HV‐LMB pouch cell, and showed its stable cycling over 100 cycles. Furthermore, the high flash point of SL significantly benefits the safety of the HV‐LMBs. Although the results in this work are only based on half cells and LMBs, they are sufficient to demonstrate the high stability of newly developed SL electrolyte toward Li metal anode and high‐voltage cathode simultaneously. More systematic evaluation of this electrolyte system regarding the FEC consumption and high‐temperature tolerance in other full‐cell system (e.g., with graphite anode) will be conducted in our future works.

## Conflict of Interest

The authors declare no conflict of interest.

## Supporting information



Supporting Information

Supporting Information Video 1

Supporting Information Video 2

Supporting Information Video 3

Supporting Information Video 4

Supporting Information Video 5

## Data Availability

The data that support the findings of this study are available in the supporting information of this article.
