# Supplementary material for: Dual‐Interphase‐Stabilizing Sulfolane‐Based Electrolytes for High‐Voltage and High‐Safety Lithium Metal Batteries
Source: Adv Sci (Weinh). 2024 Oct 1;11(44):2410129. doi: 10.1002/advs.202410129 (PMC11600293; doi:10.1002/advs.202410129)
Supplement: Supplementary file 1 — Supporting Information [file ADVS-11-2410129-s001.docx]

Supporting Information

Dual-Interphase-Stabilizing Sulfolane-Based Electrolytes for High-Voltage and High-Safety Lithium Metal Batteries

*Junhua Zhou, Chi Zhang, Huimin Wang, Yanpeng Guo, Chuan Xie, Yufeng Luo, Chao Wang, Shujing Wen, Jiehua Cai, Wancheng Yu, Fan Chen, Yufei Zhang, Qiyao Huang* and Zijian Zheng**

J. Zhou, J. Cai, W. Yu, F. Chen, Y. Zhang, Q. Huang, Z. Zheng

School of Fashion and Textiles, The Hong Kong Polytechnic University, Hong Kong SAR, China

E-mail: qi-yao.huang@polyu.edu.hk, tczzheng@polyu.edu.hk

C. Zhang, H. Wang, Y. Guo, C. Xie, Y. Luo, C. Wang, S. Wen, Z. Zheng

Department of Applied Biology and Chemical Technology, The Hong Kong Polytechnic University, Hong Kong SAR, China

Q. Huang, Z. Zheng

Research Institute for Intelligent Wearable Systems, The Hong Kong Polytechnic University, Hong Kong SAR, China

Z. Zheng

Research Institute for Smart Energy, The Hong Kong Polytechnic University, Hong Kong SAR, China

**Methods**

*Electrolyte preparation*: Chemicals for electrolyte preparation were purchased from DodoChem and used as-received, including lithium bis(trifluoromethanesulfonyl)imide (LiTFSI, 99.9%), lithium difluoro(oxalato)borate (LiDFOB, 99.9%), sulfolane (SL, 99.9%), and fluoroethylene carbonate (FEC, 99.9%). All electrolytes were prepared by dissolving the specific amount of LiTFSI salt in SL solvent with or without additives (LiDFOB, and/or FEC) in an Ar-filled glove box (H_2_O < 0.1 ppm, O_2_ < 0.1 ppm). Specifically, three kinds of electrolyte were designed, including 2 M LiTFSI in SL (LS), 2 M LiTFSI in SL with 10 vol.% FEC additives (LS-FEC), and 2 M LiTFSI in SL with 0.2 M LiDFOB and 10 vol.% FEC additives (LS-DIS).

*Electrochemical measurements*: CR2025-type coin cells were assembled with Li metal anodes (diameter, 14 mm; thickness, 400 μm; China Energy Lithium Co., Ltd.), glass fiber separators (diameter, 16.5 mm; thickness, 260 μm; Whatman, GF/A), and prepared electrolytes (80 μL) in the glove box. Charge-discharge tests were conducted under special current densities before two formation cycles at 0.1 C using a multi-range battery testing system (Neware, CT-4000) at 25 ℃. Symmetric Li||Li cells with the Li metals as both work and counter electrodes were tested with a limited capacity of 1 mAh cm^-2^ under various current densities (1 C = 1 mA cm^-2^). Asymmetric Li||Cu cells with Cu foils (diameter, 16 mm) as work electrodes were tested with a limited capacity of 1 mAh cm^-2^ and voltage of 1 V under various current densities (1 C = 1 mA cm^-2^). Li||LCO half cells with commercial LCO electrodes (diameter, 10 mm; thickness, 2.4 mAh cm^-2^; Guangdong Canrd New Energy Technology Co., Ltd.) as work electrodes were tested with a voltage range of 3~4.65 V, 3~4.7 V, and 3~4.75 V under various current densities (1 C = 220 mA g^-1^).

Cyclic voltammetry (CV) and electrochemical impedance spectra (EIS) were collected using an electrochemical workstation (BioLogic, VMP3). The CV tests based on Li||Cu and Li||Al cells were used to evaluate the reduction and oxidation stability of prepared electrolytes with a scan rate of 0.5 mV s^-1^, and a potential range of -0.2~3 V and 3~6 V respectively. The electrochemical reaction mechanism of LCO was explored by testing the CV of Li||LCO cells with a scan rate of 0.5 mV s^-1^, in the potential range of 3~4.65 V, 3~4.7 V, and 3~4.75 V. The impedance of SEI and CEI were obtained by EIS tests of cycled Li||Cu and Li||LCO cells in the frequency range of 10 mHz~7 MHz with a potential amplitude of 5 mV. The Li^+^ conductivity and activation energy of SL-based electrolytes were acquired by EIS using SS||SS cells (SS represents stainless steel) at 30, 40, 50, and 60 ℃. Li||Al cells were tested with the potentiostatic mode at 4.75 V for 24 h to assess the Al foil corrosion characteristic of different kinds of electrolytes.

Pouch cells were assembled in the glove box as follows. First, the Ni tab and Al tab were stuck onto a thin Li foil (thickness, 50 μm; size, 4 × 6 cm^2^) and thick LCO cathode (thickness, 4.2 mAh cm^-2^; size, 4 × 6 cm^2^) respectively. The cathode, separator with Al_2_O_3_ coating layer (Guangdong Canrd New Energy Technology Co., Ltd.), and anode were then stacked layer-by-layer to make a jellyroll cell. After injecting the LS-DIS or commercial ester-based electrolyte (EE, 1M LiPF_6_ in DMC: EC = 2:1 wt.%, battery grade) with the amount of 3 g Ah^-1^, the cell was sealed by Al laminated films. The cycling performance of the pouch cell was tested with a voltage range of 3~4.65 V at 0.1 C (1 C = 220 mA g^-1^). To evaluate the thermal stability of electrolytes at high voltage, pouch cells were charged to 5 V, and infrared thermal images were collected by an infrared camera (FLIR T1010) synchronously.

For in situ optical observation, specially designed Li||Li cells with glass windows were assembled in the glove box. The cells were then discharged at a current density of 1 mA cm^-2^ for 1 h, and lithium dendrite growth videos were collected by optical microscopy (Andonstar, DP-5) synchronously.

*Materials characterization*: The solvation structures of prepared electrolytes were evaluated by Raman spectra. The electrolytes were sealed in quartz cuvettes to avoid air exposure and then tested through a Renishaw Micro-Raman Spectroscopy System using a laser source with a wavelength of 785 nm. The thermal stability and low-temperature character of electrolytes were measured by a thermogravimetric analyzer (TGA, Mettler) under N_2_ protection, and a differential scanning calorimeter (DSC, Mettler) respectively. The burning capability of electrolytes was evaluated by a flame test using glass fiber separators infiltrated with electrolytes.

For failure analyses, cells after cycling were disassembled in the glove box to obtain working electrodes, which were then washed with DMC solvents, and dried at room temperature. Thereafter, the electrodes were transferred out from the glove box for different kinds of measurements with special holders to avoid air exposure (e.g., XRD holders sealed with PI tapes, and XPS vacuum transfer chambers). XRD patterns were collected using a Rigaku SmartLab Advance diffractometer with a 9 kW high-power X-ray generator. SEM images and EDX spectra were obtained using a field emission SEM (Tescan MAIA3) equipped with an EDX detector operating at 15 kV and 15 mA. XPS patterns were collected by applying a Thermo Fisher Scientific Nexsa XPS instrument with an Al Kα radiation source. Binding energy in this work was calibrated based on carbon contamination with the C1s peak at 284.8 eV. Raman spectra were measured through the Raman system using a laser source with a wavelength of 532 nm. Conventional TEM images were captured using field emission TEM (JEOL JEM-2100F) operating at 200 kV.

For cryogenic transmission electron microscope (cryo-TEM) characterization, Li||Cu cells were assembled with the TEM Cu grids as working electrodes. After 30 minutes of Li metal deposition with a current density of 2 mA cm^-2^, the cells were disassembled to obtain the Cu grids, which were then washed with DMC, and dried at room temperature in the glove box. The grids were transferred with a vacuum transfer chamber and thereafter handled with a cryo-autoloader system. Cryo-TEM images were collected using the Thermo Fisher Krios G4 Cryo-TEM operating at 300 kV in an ultra-low temperature (78 K) and dose of electrons (~8 e Å^-2^ s^-1^ × 10 s). The Cryo-TEM was equipped with zero-loss filtering of the Selectris Filters and Falcon 4i direct electron detector, which could further enhance image resolution.

*Density functional theory calculations*: All density functional theory calculations were performed using the ORCA 5.0 package. Geometrical optimization and frequency calculations are conducted with m062x^[1]^ functional and def2-TZVP^[2, 3]^ basis. Dispersion correction is employed through Grimme’s D3 damping function.^[4]^


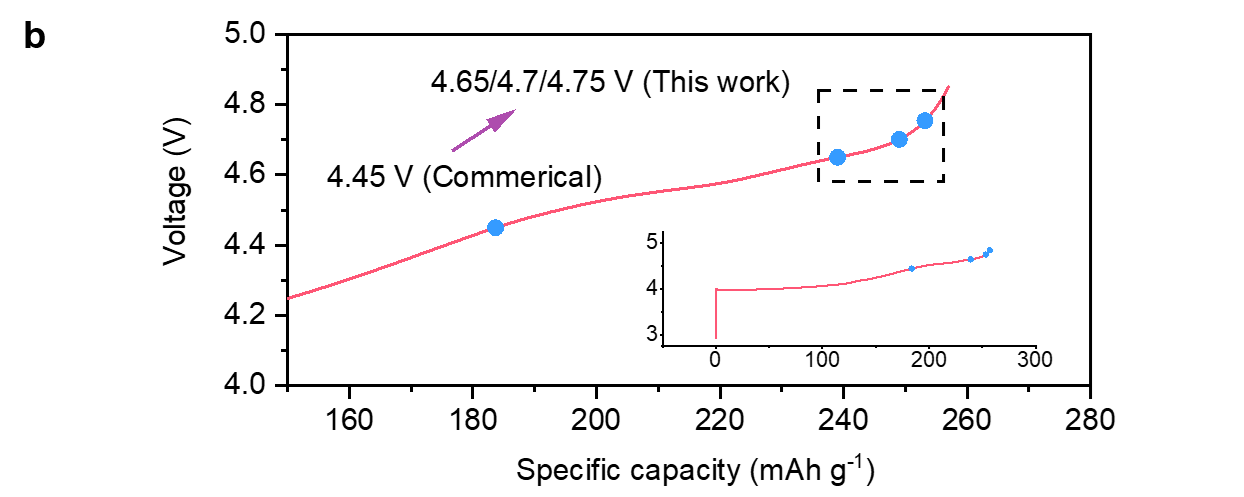


**Figure S1.** The typical charge curve of LCO showing the advantages of improving the cut-off voltage.


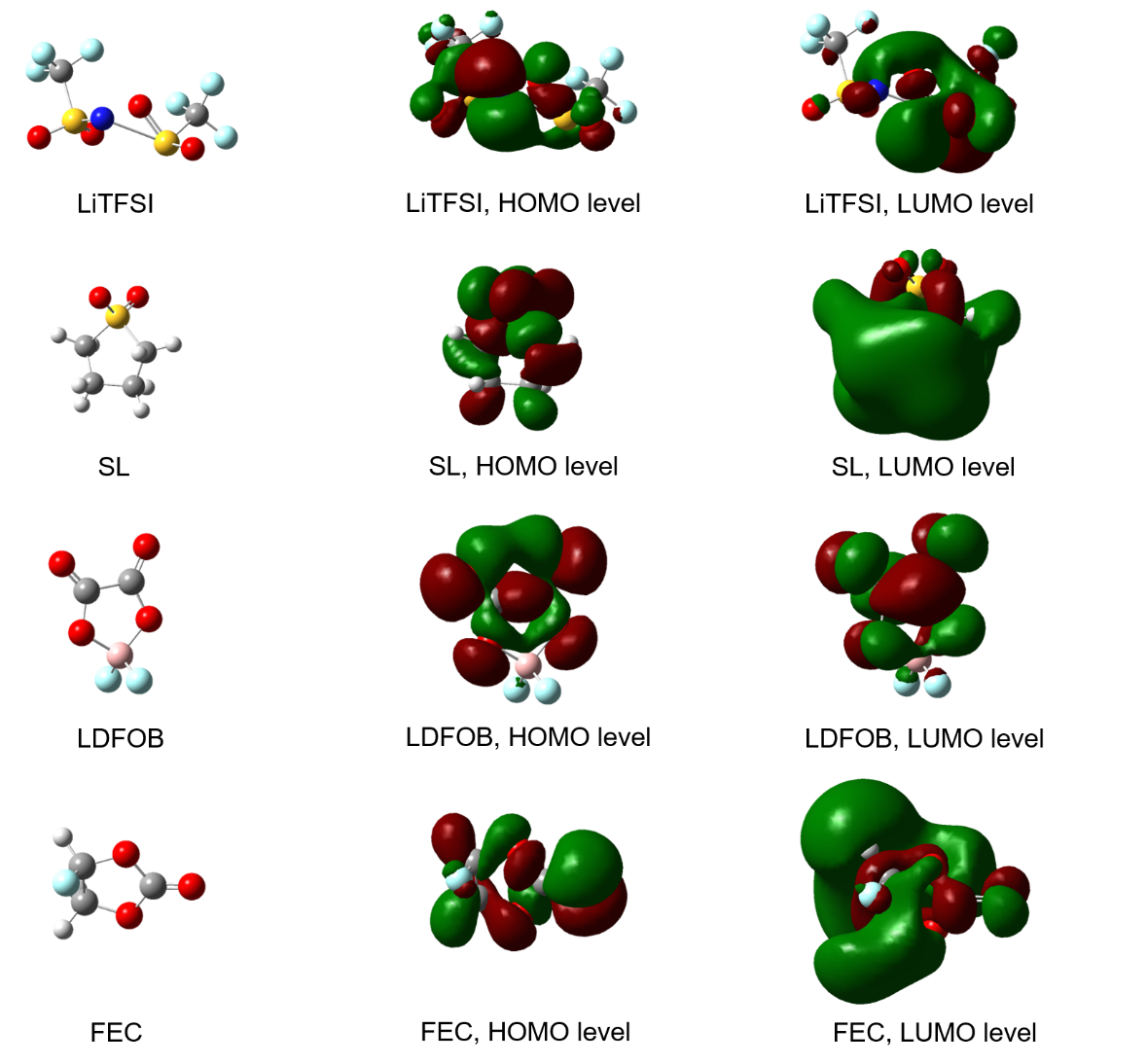


**Figure S2.** The HOMO and LUMO energy level diagrams of compounds used for electrolyte preparation.


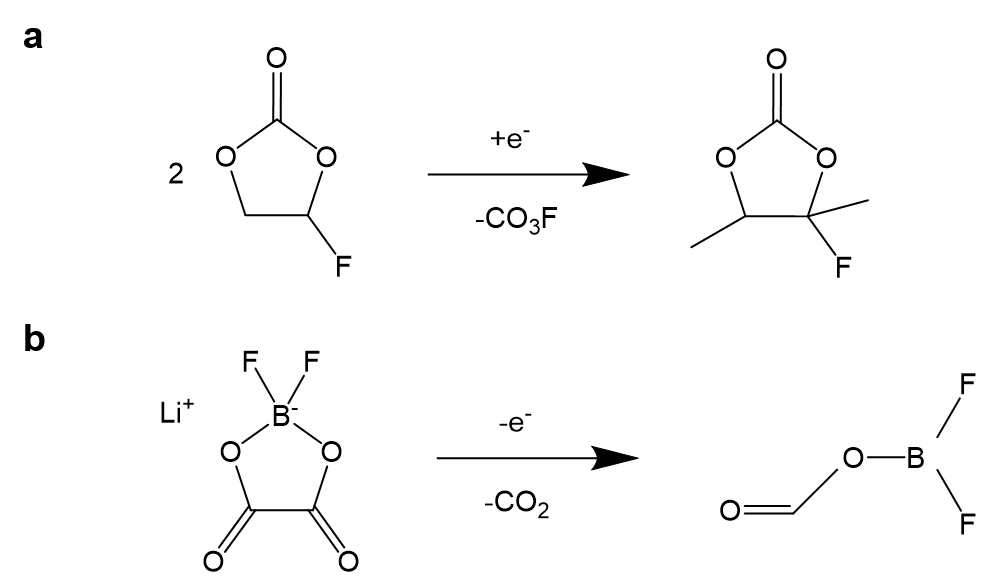


**Figure S3.** Proposed mechanism for (**a**) the reduction of FEC and (**b**) oxidation of LiDFOB.


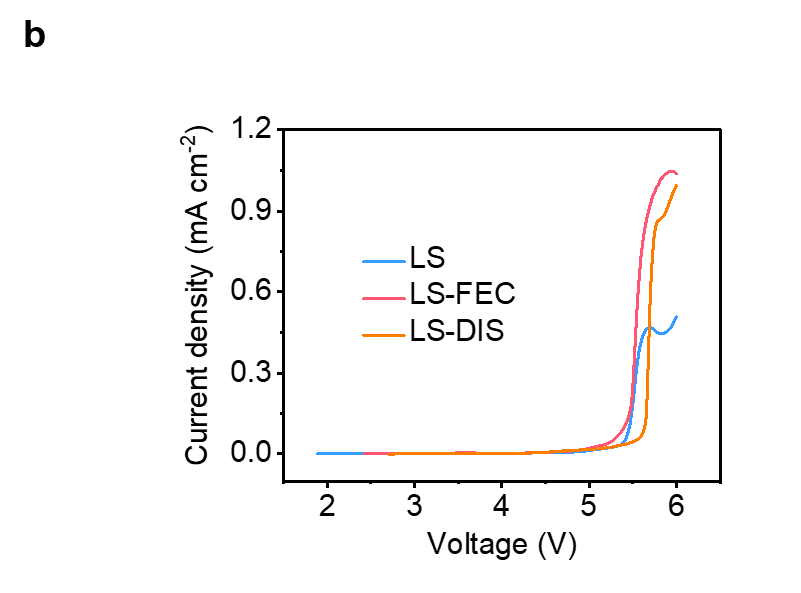


**Figure S4.** LSV curves of Li-Al cells from open circuit voltage to 6 V at a scan rate of 0.5 mV s^-1^ in various electrolytes. All electrolytes show a high oxidation potential of more than 5 V vs. Li/Li^+^, attributed to the extremely low HOMO of the SL solvent.


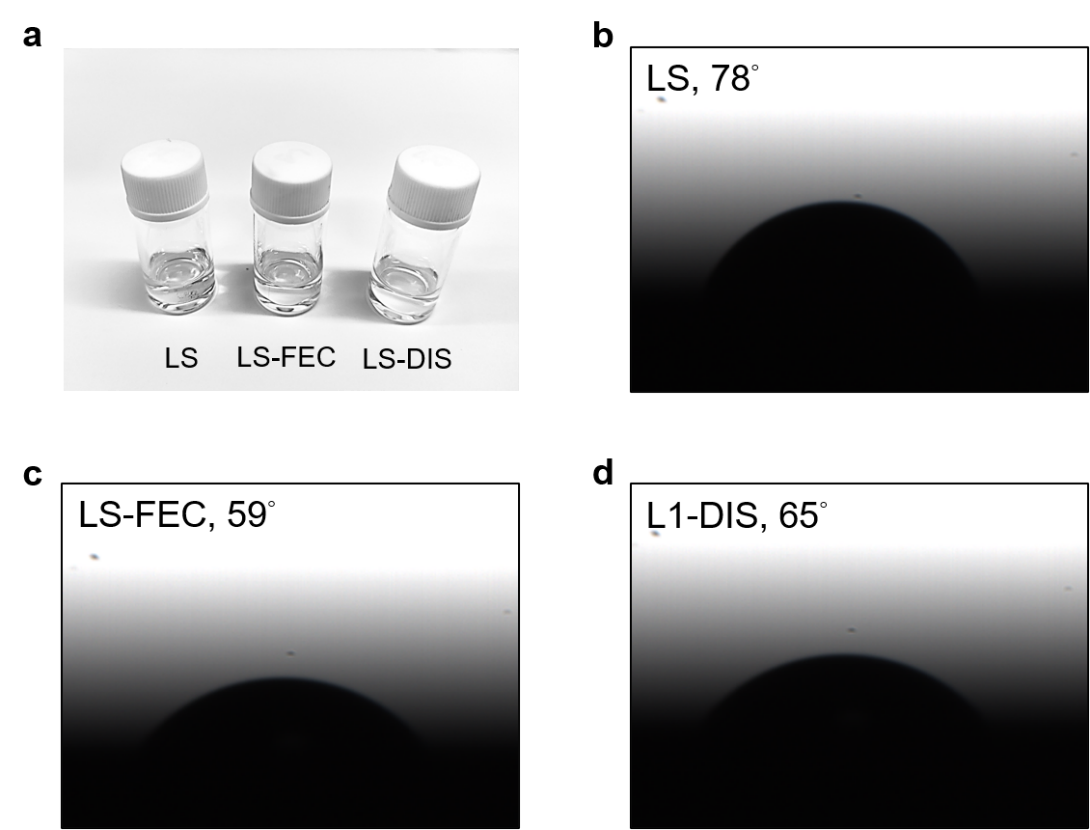


**Figure S5.** (**a**) Pictures of prepared electrolytes. (**b-d**) Contact angle results of various electrolytes.


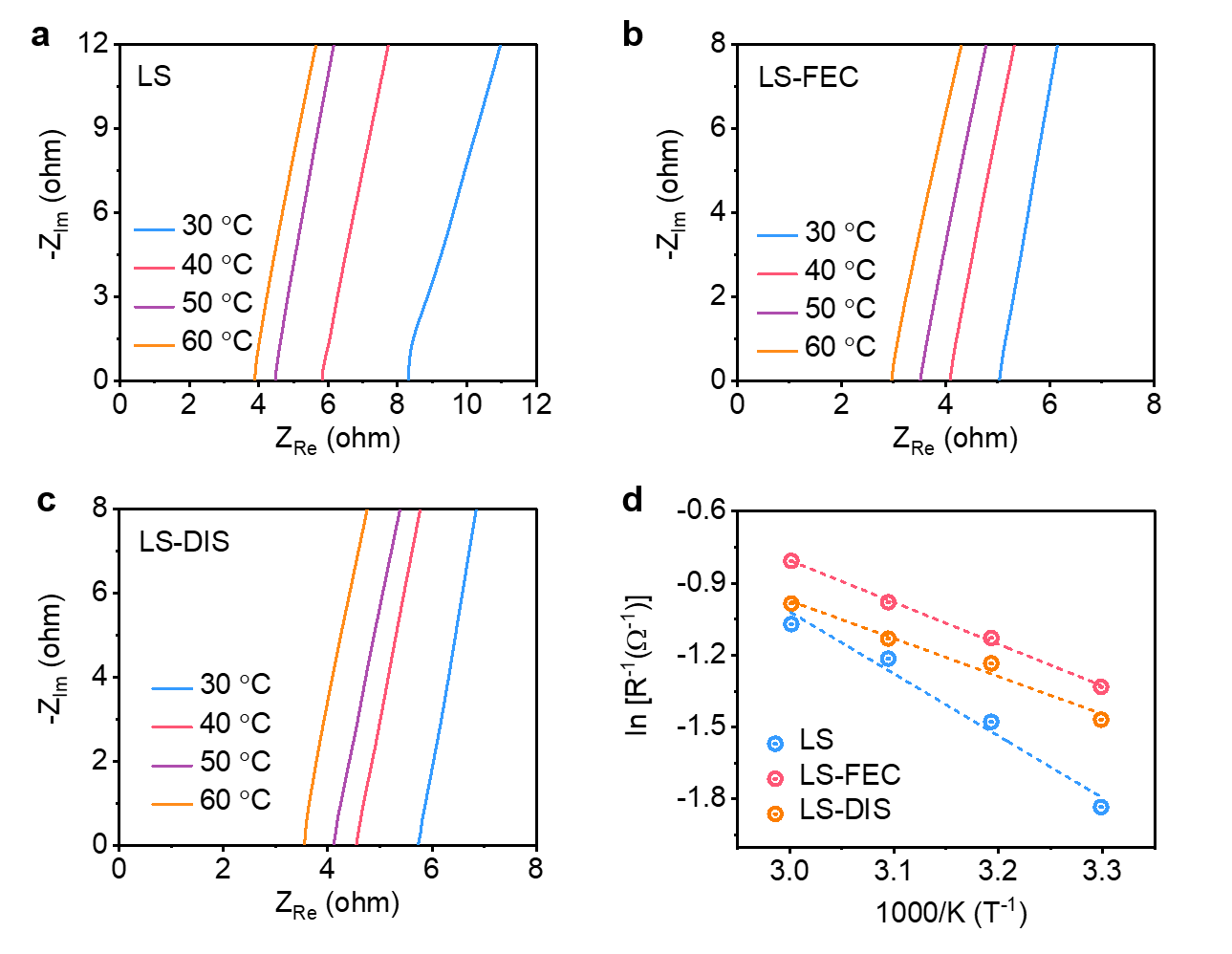


**Figure S6.** EIS curves of SS-SS cells in the (a) LS, (b) LS-FEC, and (c) LS-DIS at various temperatures. (d) Activation energies Li^+^ diffusion in corresponding electrolytes.


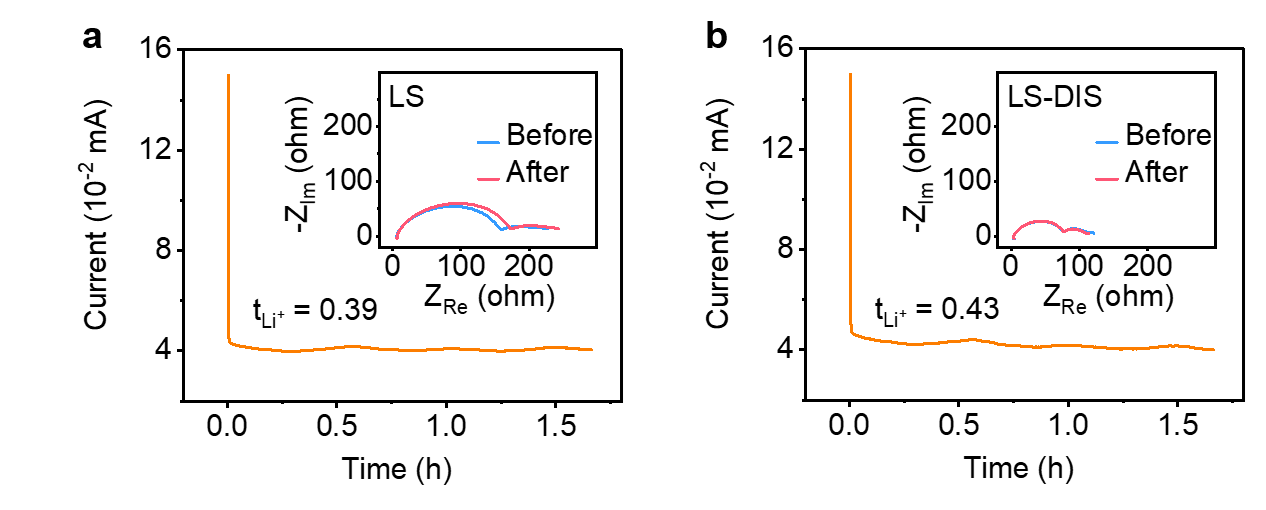


**Figure S7.** Chronoamperometry profiles of Li||Li cells under the polarization voltage of 10 mV and the corresponding EIS before and after polarization to obtain the lithium transference number (t_Li+_) in the (**a**) LS and (**b**) LS-DIS.


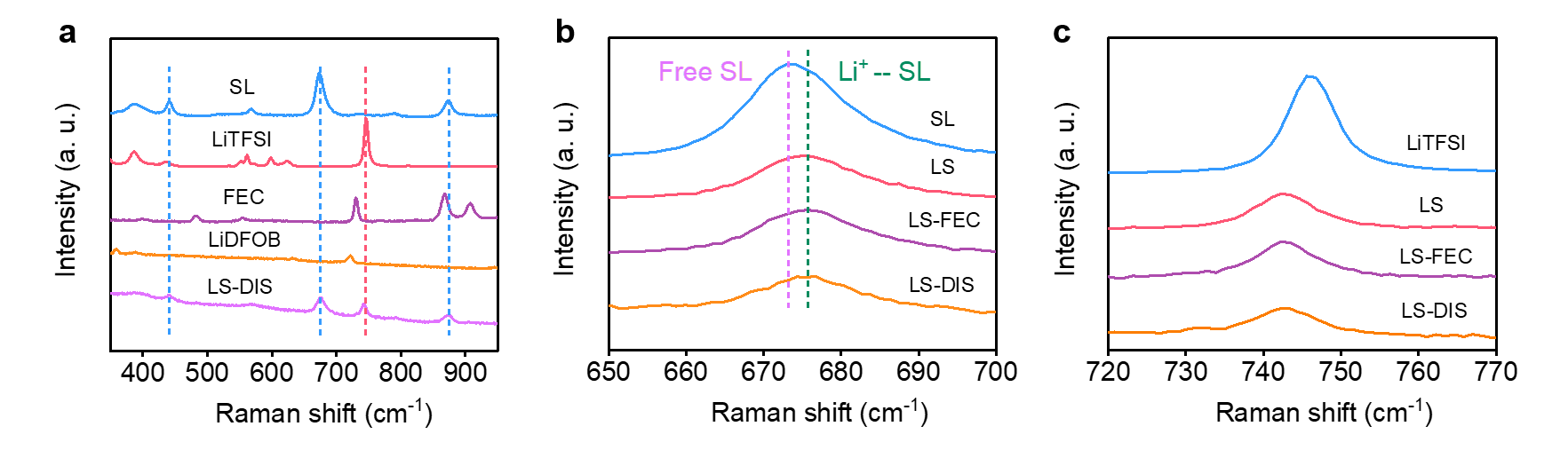


**Figure S8.** (**a**) Raman curves of compounds used for electrolyte preparation, and (**b**, **c**) electrolytes with various additives. In the **a**, the peaks of LS-DIS are mainly attributed to the LiTFSI and SL. No peak is found in the LS-DIS that can be indexed to the FEC and LiDFOB, since the amount of used additives is tiny. In **b** and **c**, the identical peak positions of LS, LS-FEC, and LS-DIS indicate the additives do not alter the solvation structure obviously.


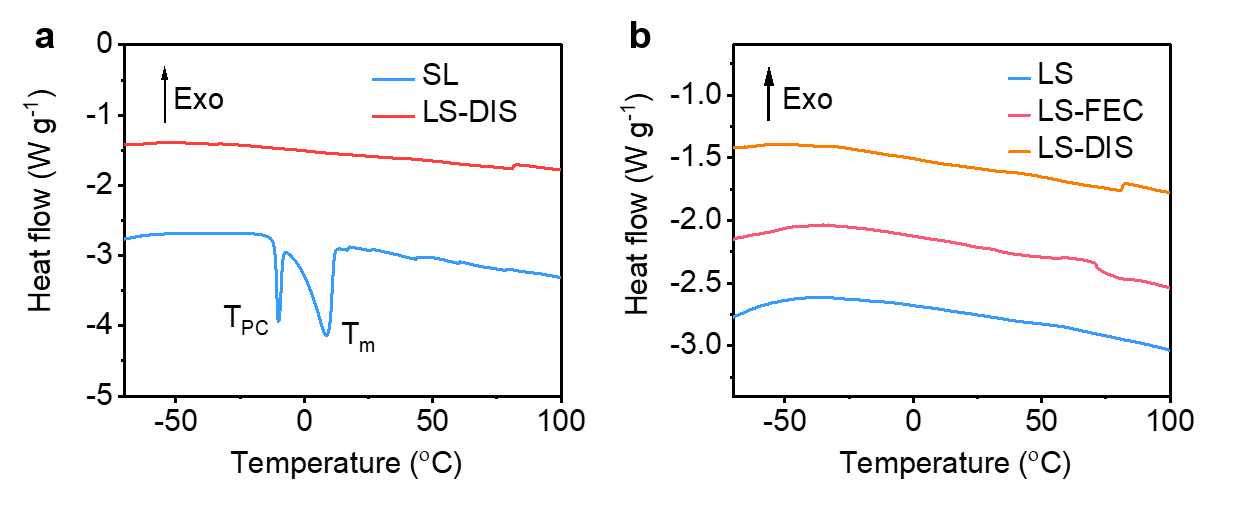


**Figure S9.** (**a, b**) DSC curves of the SL solvent and various electrolytes. The disappeared plastic crystal temperature (T_pc_) and melting point temperature (T_m_) demonstrate the prolonged liquid range of SL after introducing Li salt and additives.


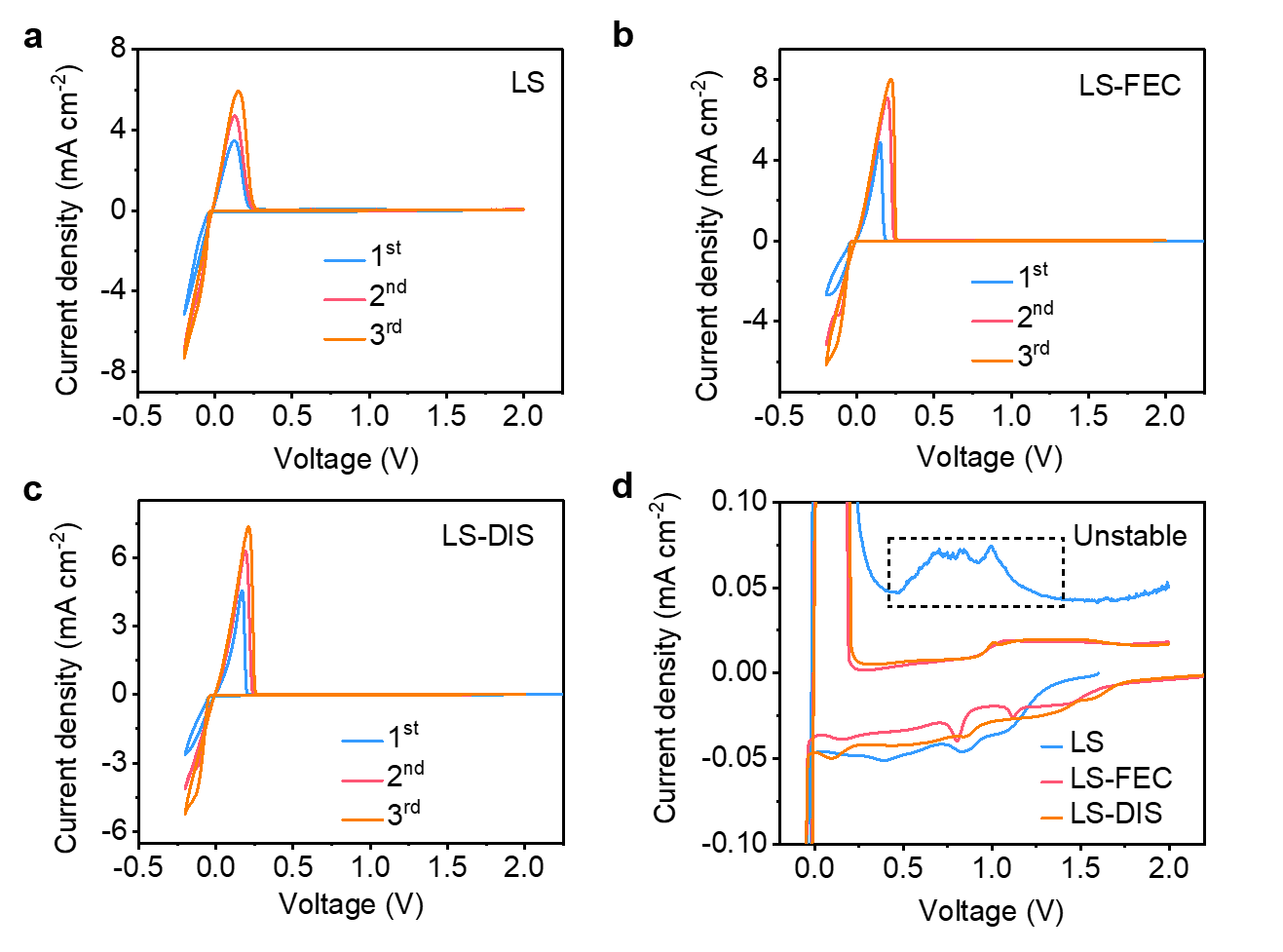


**Figure S10.** CV curves of Li||Cu cells with a scan rate of 0.5 mV s^-1^ in the (**a**) LS, (**b**) LS-FEC, (**c**) LS-DIS, and (**d**) enlarged figures. The curve highlighted with the dashed rectangle indicates the incompatibility between Li metal and the LS.


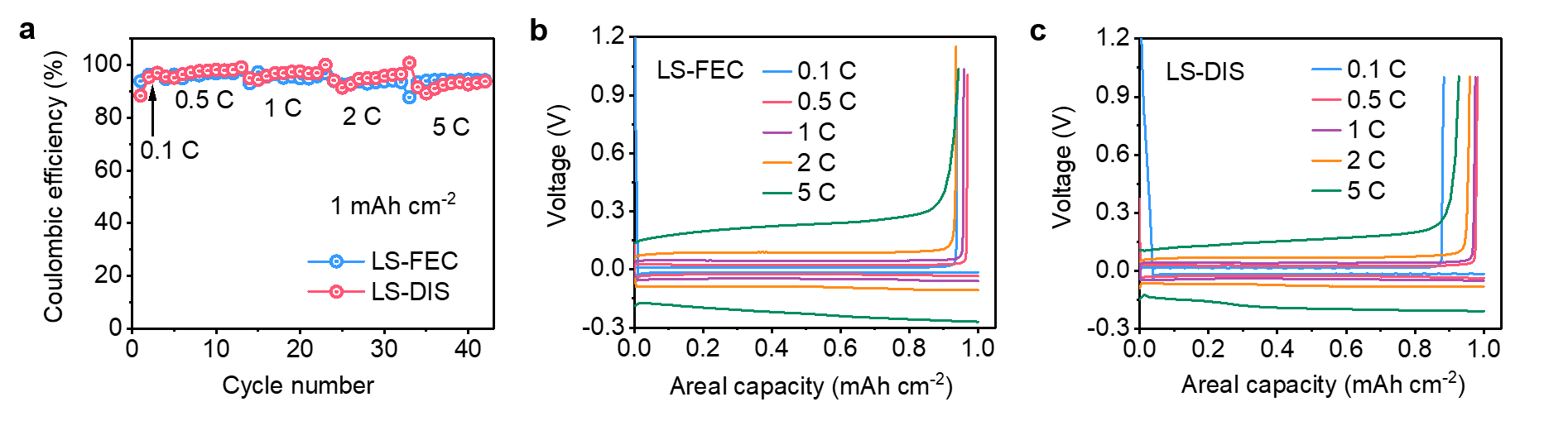


**Figure S11.** (**a**) Coulombic efficiency and (**b**, **c**) corresponding charge-discharge curves of Li||Cu cells in the LS-FEC and LS-DIS at various current densities (1 C =1 mA g^-1^).


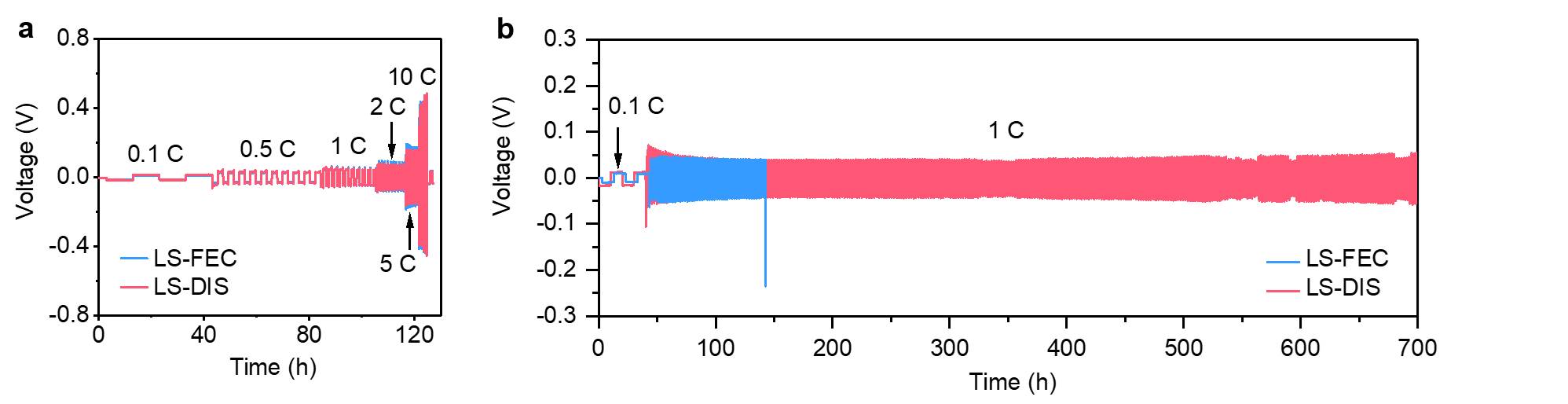


**Figure S12.** (**a**) Voltage profiles at various current densities and (**b**) cycling patterns at 1 C of Li||Li cells in the LS-FEC and LS-DIS.


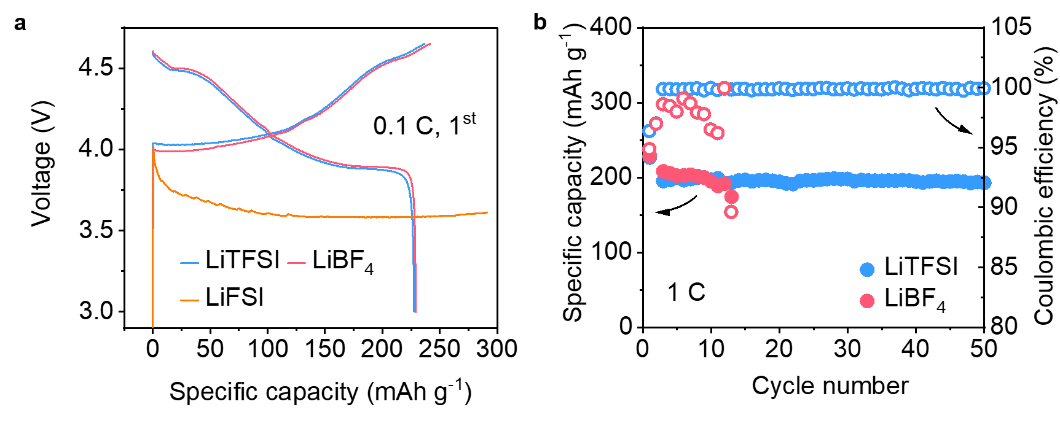


**Figure S13.** (**a, b**) Charge-discharge curves and corresponding cycling performance of high-voltage LCO in the LS-DIS with various Li salts.


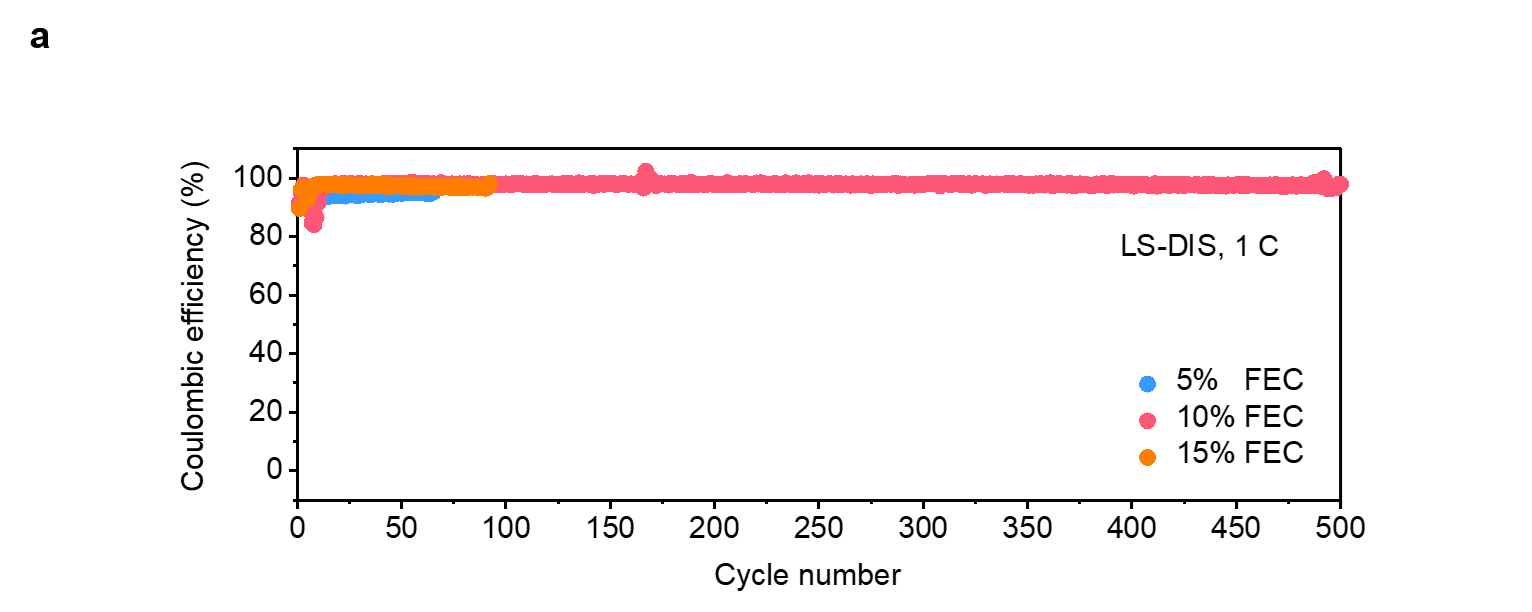


**Figure S14.** Coulombic efficiency at 1 C of Li||Cu cells in the LS-DIS with various ratios of FEC. The LS-DIS with 10% FEC shows slightly higher Coulombic efficiency than the 5% case, whereas further increasing the content of FEC cannot enhance the efficiency obviously. Therefore, the optimum amount of FEC additive in the LS-DIS was set as 10% in volume. Moreover, the moderate concentration of LiTFSI, 2 M, was chosen, which avoids the drawbacks of using high-concentration Li salts. Finally, the saturation concentration of LiDFOB in the 2 M LiTFSI in SL, 0.2 M, was chosen to elevate the Coulombic efficiency of the high-voltage LCO cathode.


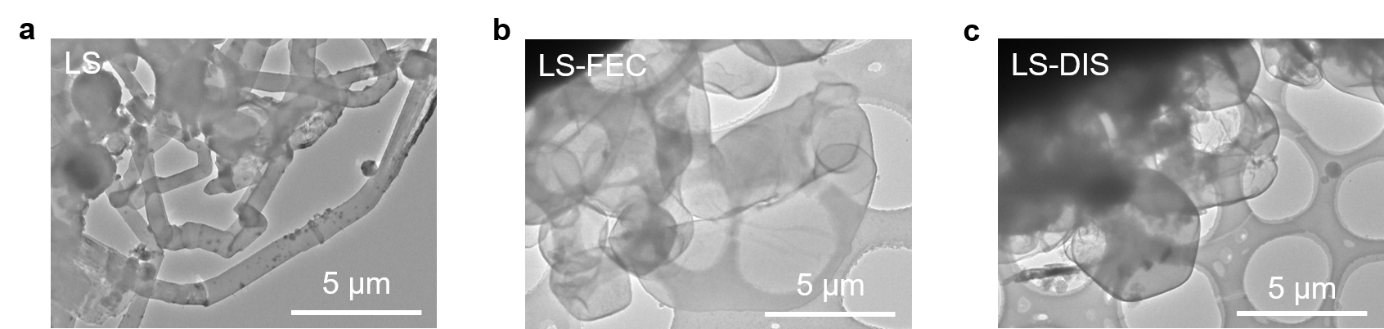


**Figure S15.** Cryo-TEM images of Li metal in the (**a**) LS, (**b**) LS-FEC, and (**c**) LS-DIS.


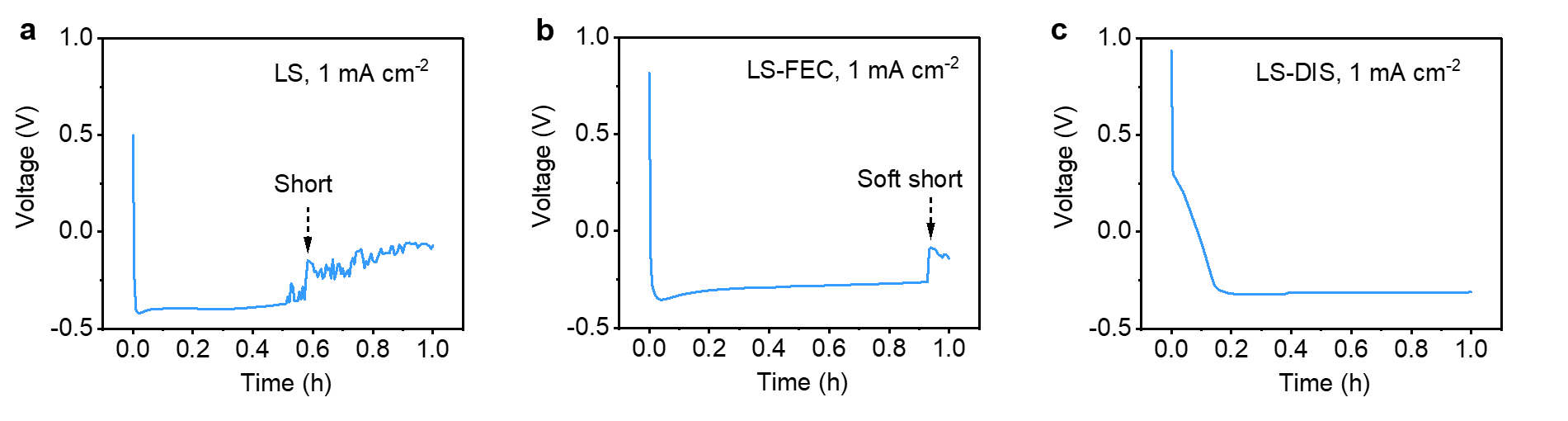


**Figure S16.** Voltage profiles of Li||Li cells with various electrolytes for in-situ optical microscopy tests.


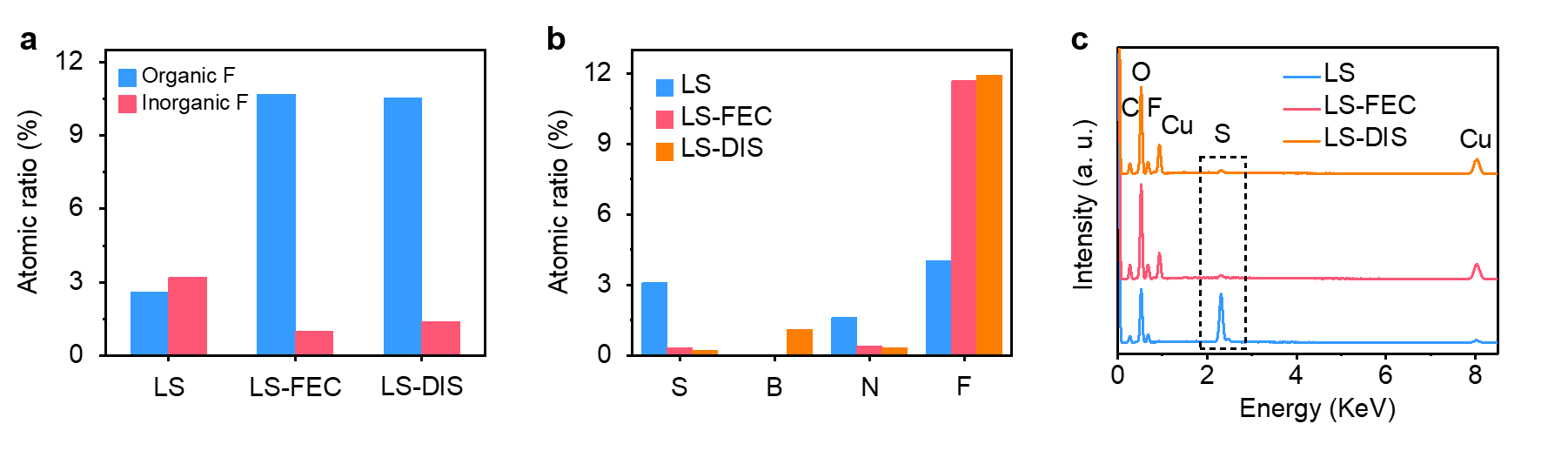


**Figure S17.** (**a**) The F species is composed of the organic F (C-F) and inorganic F (Li-F). (**b**) Atomic ratio showing the SEI composition derived from various electrolytes based on XPS tests. (**c**) EDS patterns of Li metal in various electrolytes. The large amounts of S species in the LS mainly originate from the decomposition of SL, which indicates the incompatibility between Li metal and the LS.


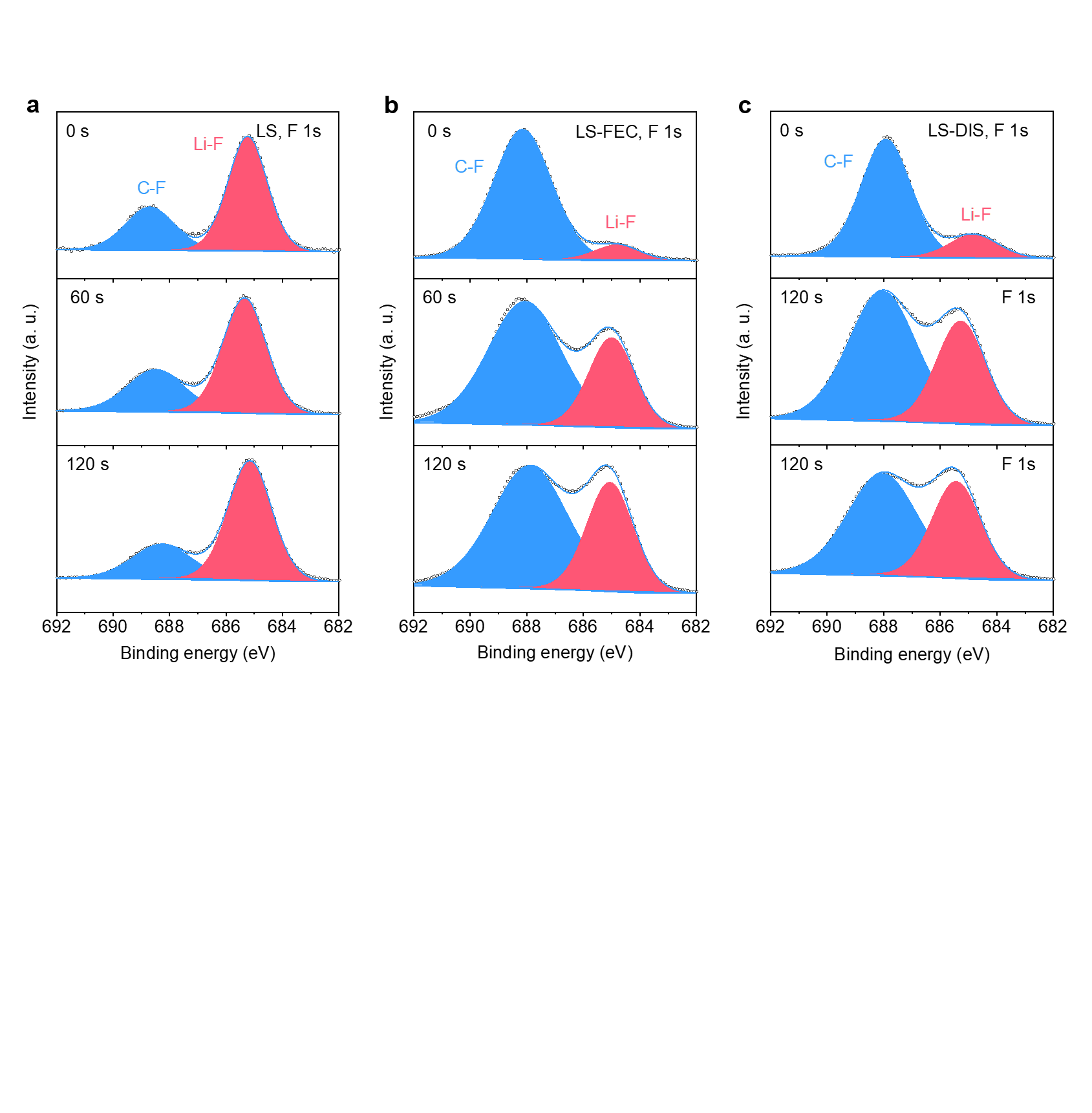


**Figure S18.** F 1s XPS patterns with etching conditions of Li metal after 50 cycles in various electrolytes.


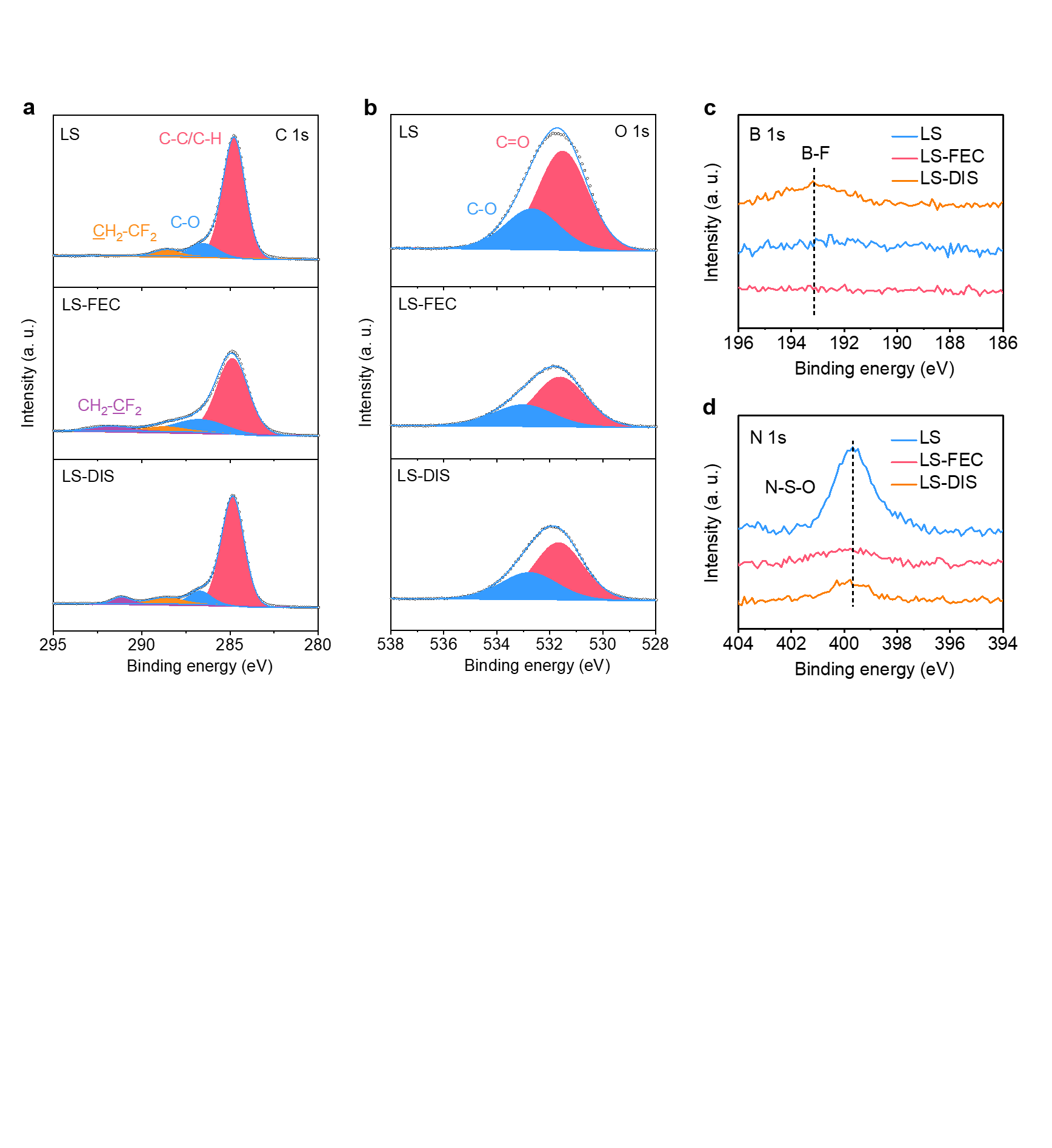


**Figure S19.** (**a**) C 1s, (**b**) O 1s, (**c**) B 1s, and (**d**) N 1s XPS patterns of Li metal with various electrolytes.


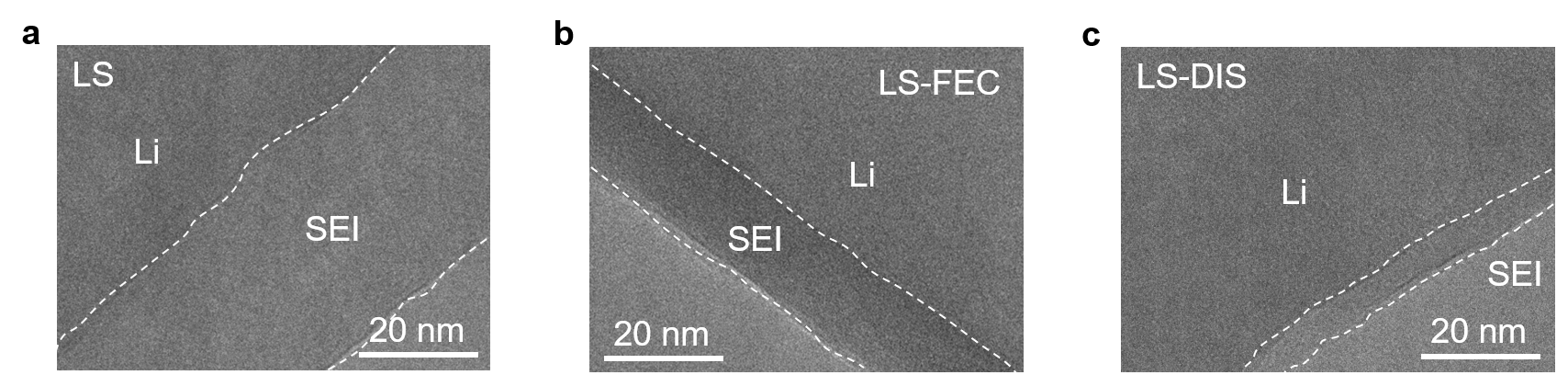


**Figure S20.** Cryo-TEM images of Li metal in the (**a**) LS, (**b**) LS-FEC, and (**c**) LS-DIS.


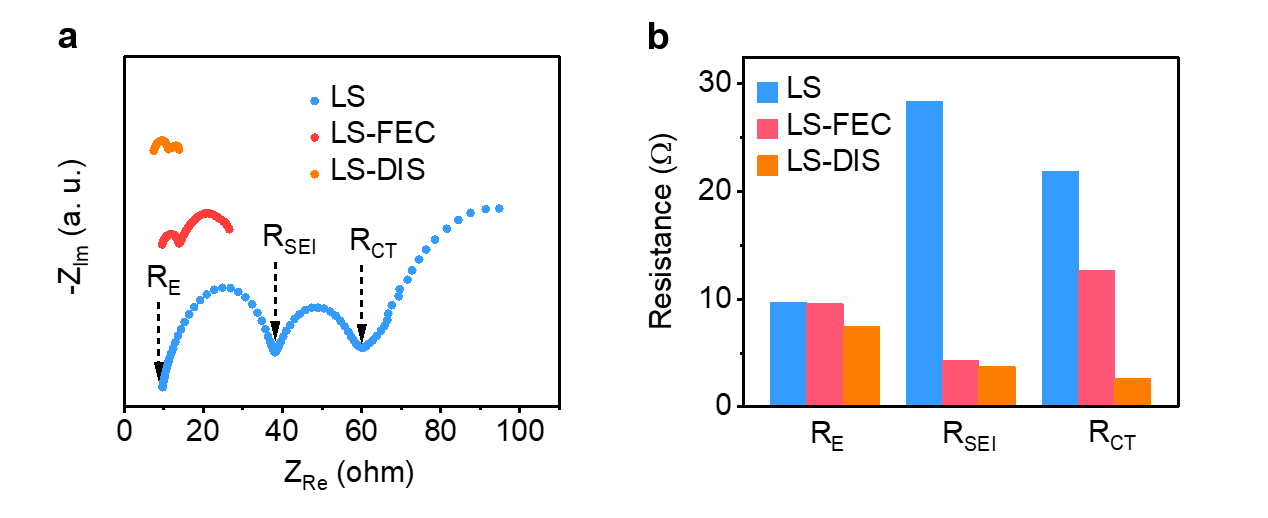


**Figure S21.** (**a**) EIS patterns and corresponding (**b**) resistance value of Li||Cu cells with various electrolytes.


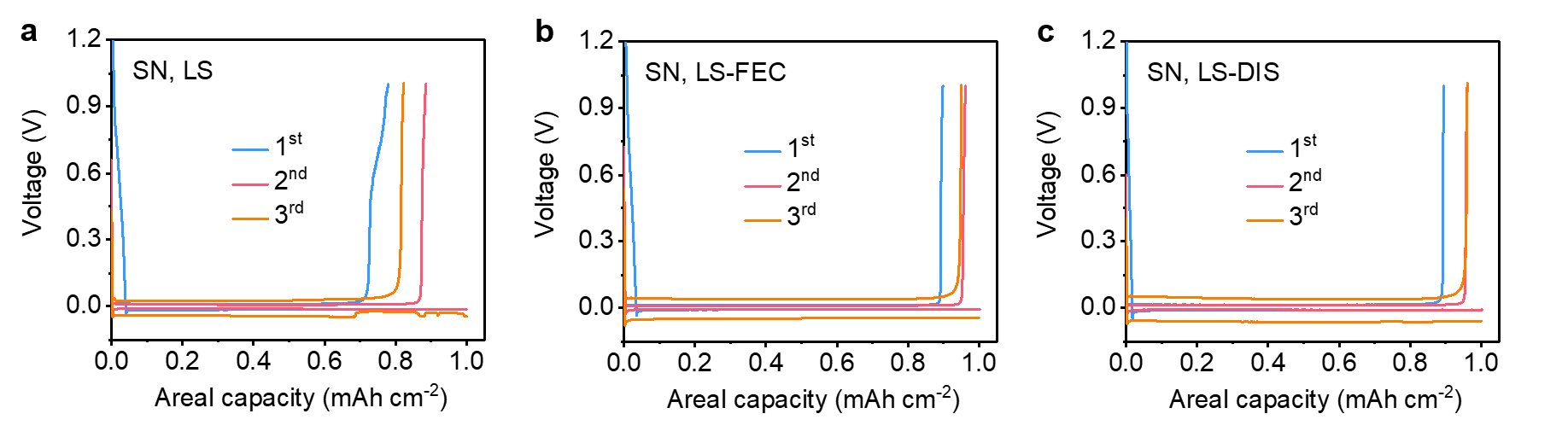


**Figure S22.** Charge-discharge curves of Li||Cu cells at 0.1 C with various SN-based electrolytes, including the (**a**) LS, (**b**) LS-FEC, and (**c**) LS-DIS. The FEC additive is also necessary for the stable operation of Li metal in SN-based electrolytes.


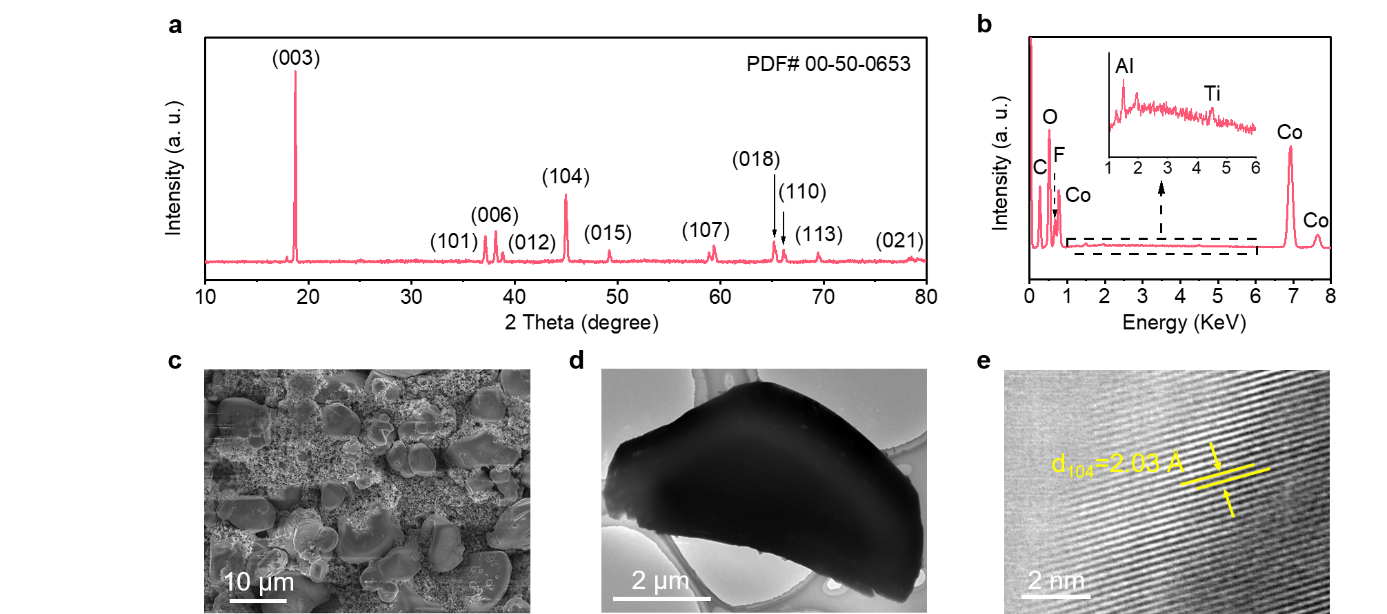


**Figure S23.** (**a, b**) XRD and EDS spectra of pristine LCO. (**c-e**) SEM and TEM images of pristine LCO.


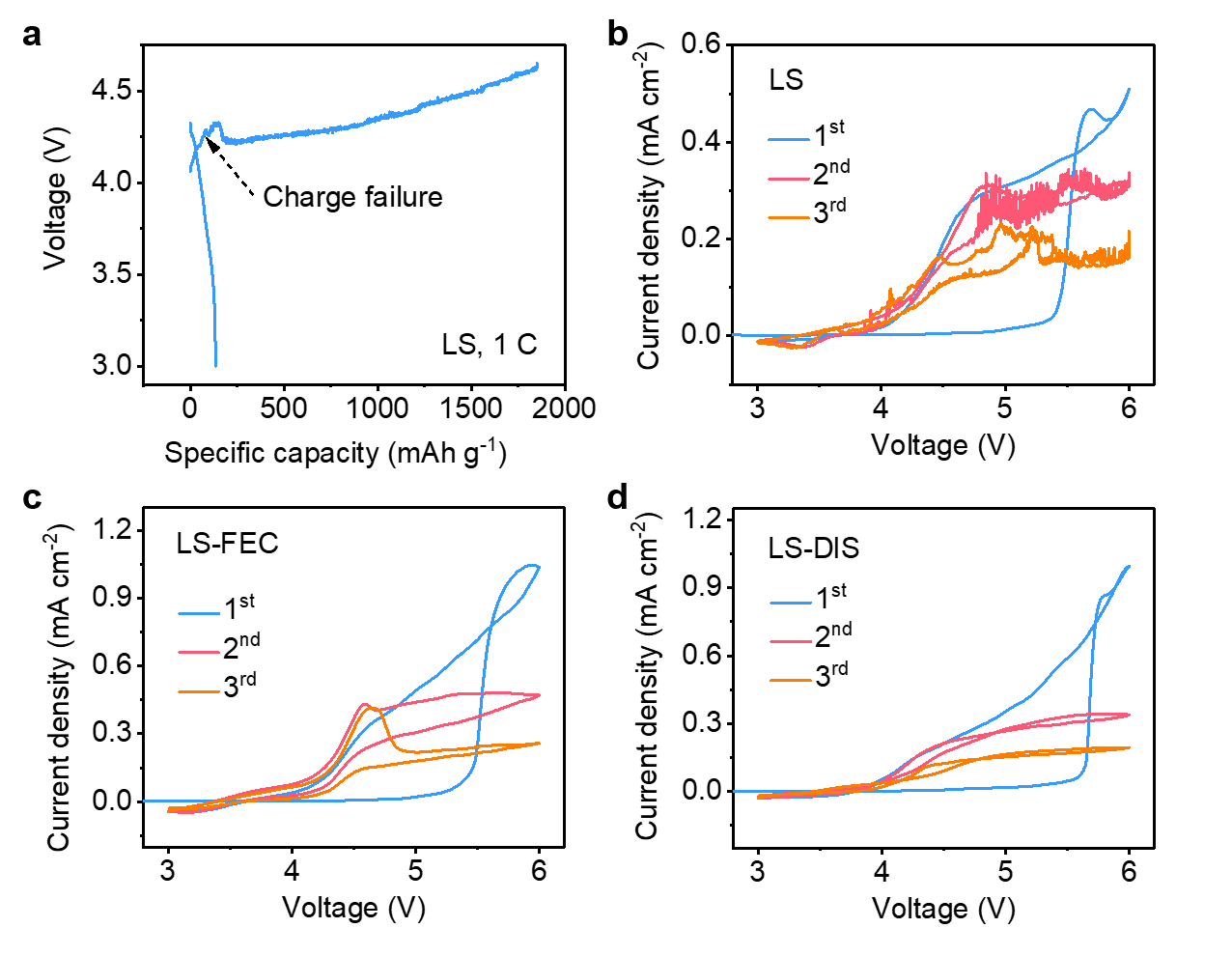


**Figure S24.** (**a**) Charge-discharge curves of LCO cathode at 1 C (1 C = 220 mA g^-1^) with a voltage range of 3~4.65 V in the LS. The LCO in the LS without the additives cannot normally charge, which originates from the Al corrosion. CV curves of Li||Al cells with a scan rate of 0.5 mV s^-1^ for the initial three cycles in the (**b**) LS, (**c**) LS-FEC, and (**d**) LS-DIS. The Al foil in the LS without the additives shows burrs, which originate from the Al corrosion.


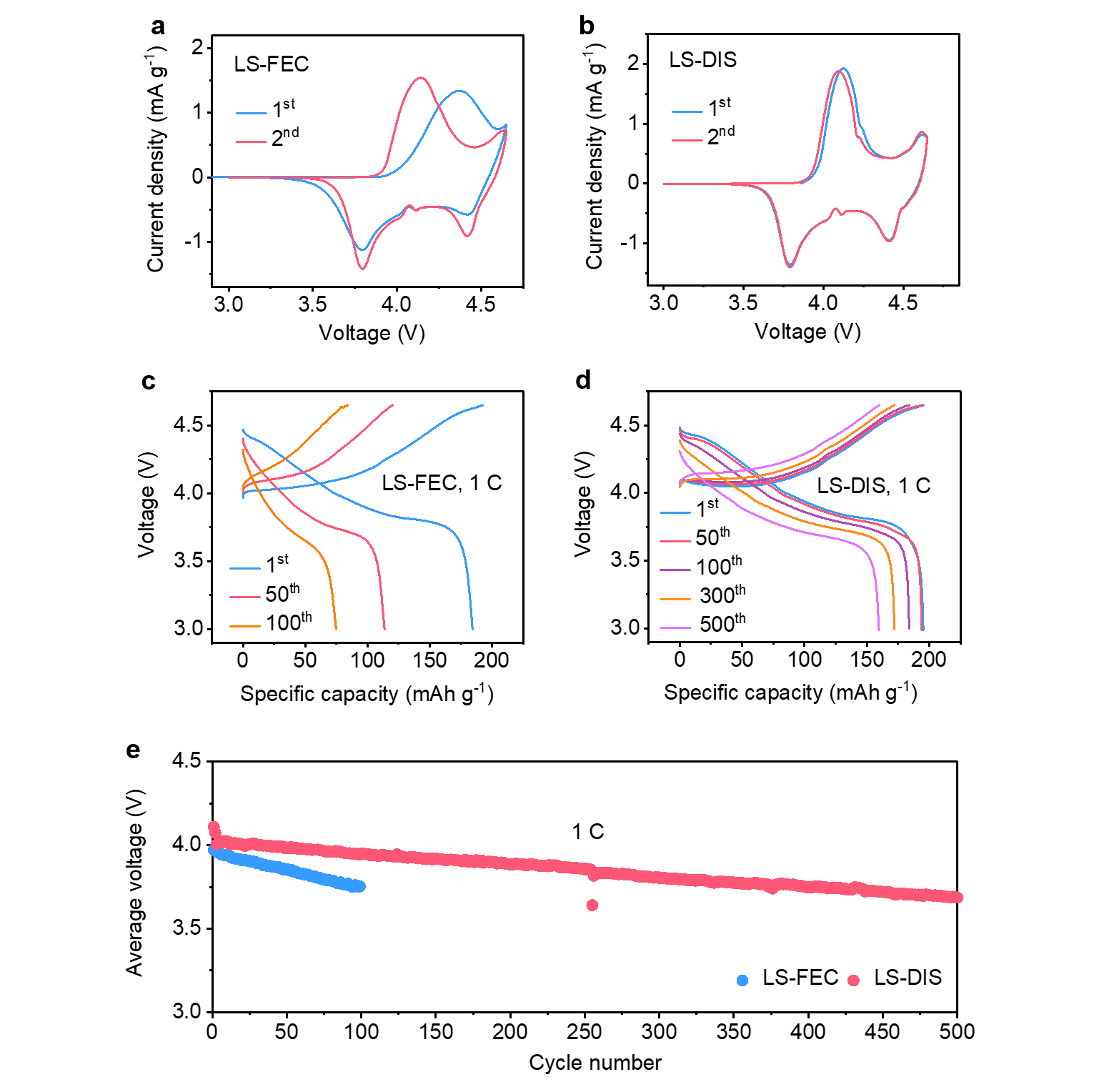


**Figure S25.** CV curves of LCO cathode in the (**a**) LS-FEC and (**b**) LS-DIS with a scan rate of 0.5 mV s^-1^. (**c**, **d**) Charge-discharge curves and (**e**) average voltage plots of LCO cathode with a voltage range of 3~4.65 V at 1 C in the LS-FEC and LS-DIS.


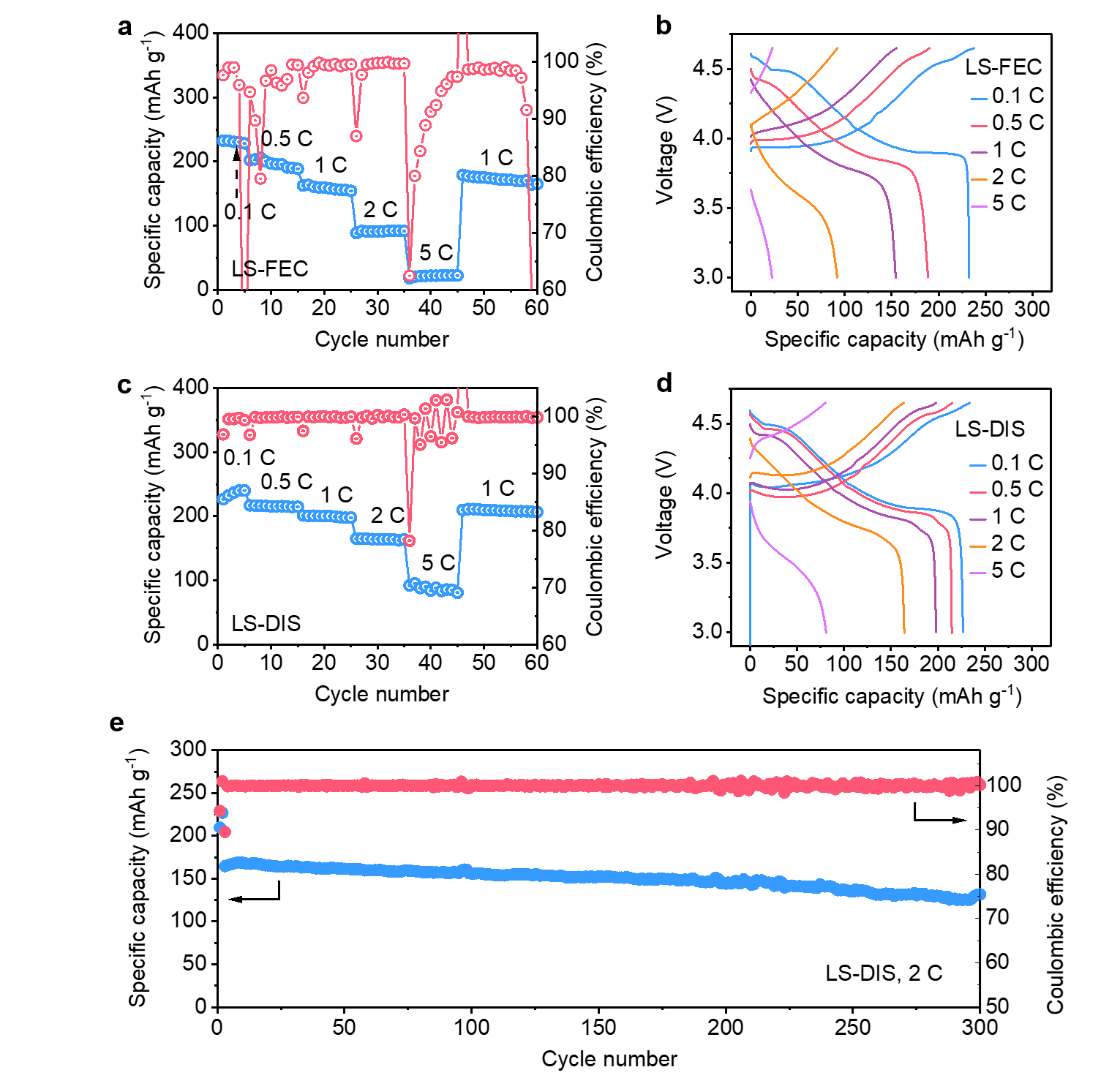


**Figure S26.** (**a**, **c**) Rate performance, (**b**, **d**) corresponding charge-discharge curves, and (**e**) cycling performance at 2 C of LCO cathode with a voltage range of 3~4.65 V in the LS-F and LS-DIS.


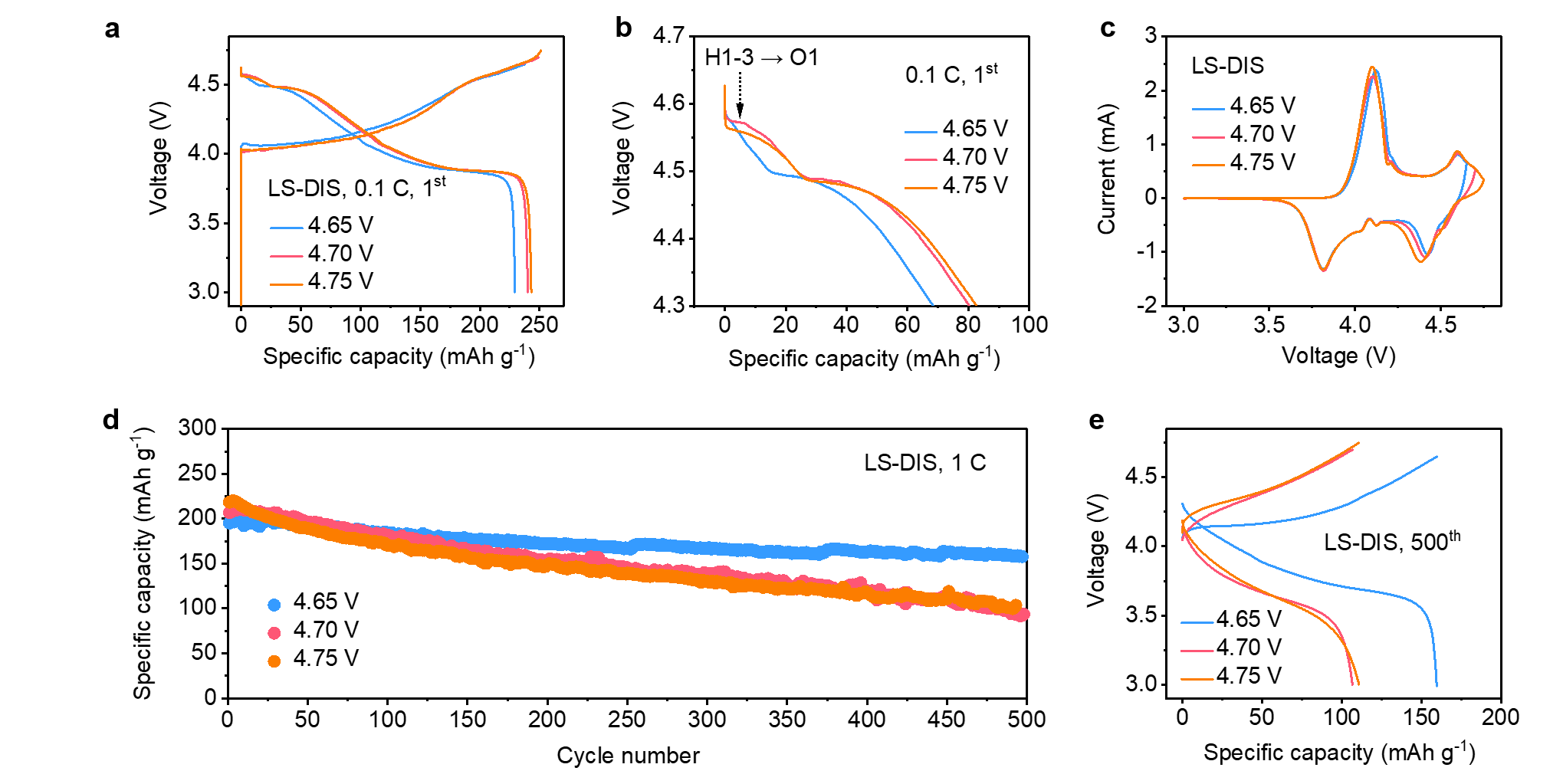


**Figure S27.** Electrochemical performance of LCO in the LS-DIS with a voltage range of 3~4.65 V, 3~4.70 V, and 3~4.75 V. (**a, b**) The initial charge-discharge curves at 0.1 C and corresponding enlarged plots. (**c**) CV curves with a scan rate of 0.5 mV s^-1^. (**d, e**) Cycling performance and corresponding charge-discharge curves at 1 C after 500 cycles.


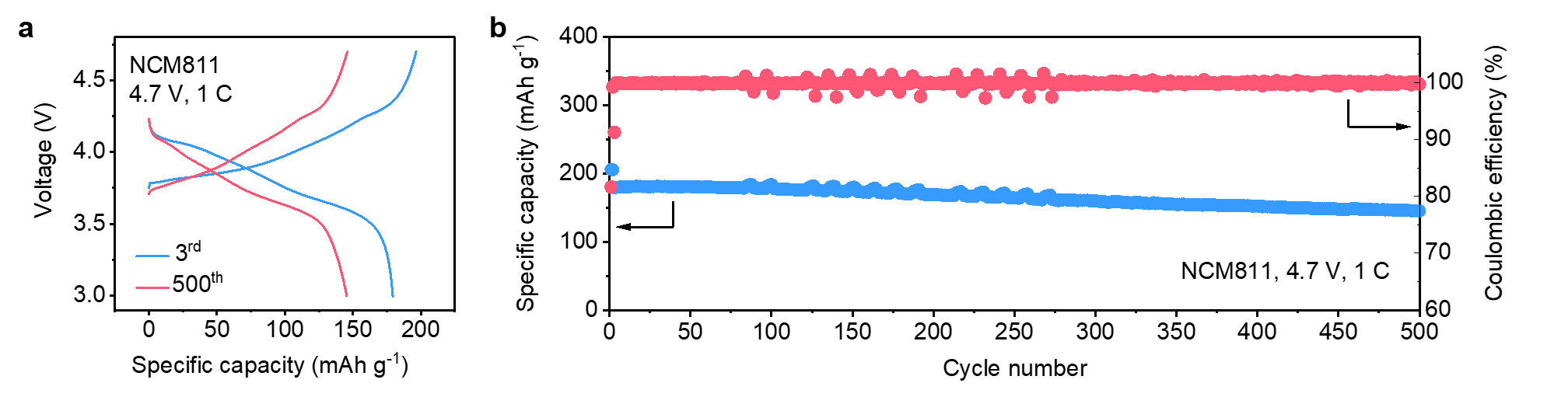


**Figure S28.** (**a, b**) Charge-discharge curves and corresponding cycling performance of high-voltage NCM811 in the LS-DIS.


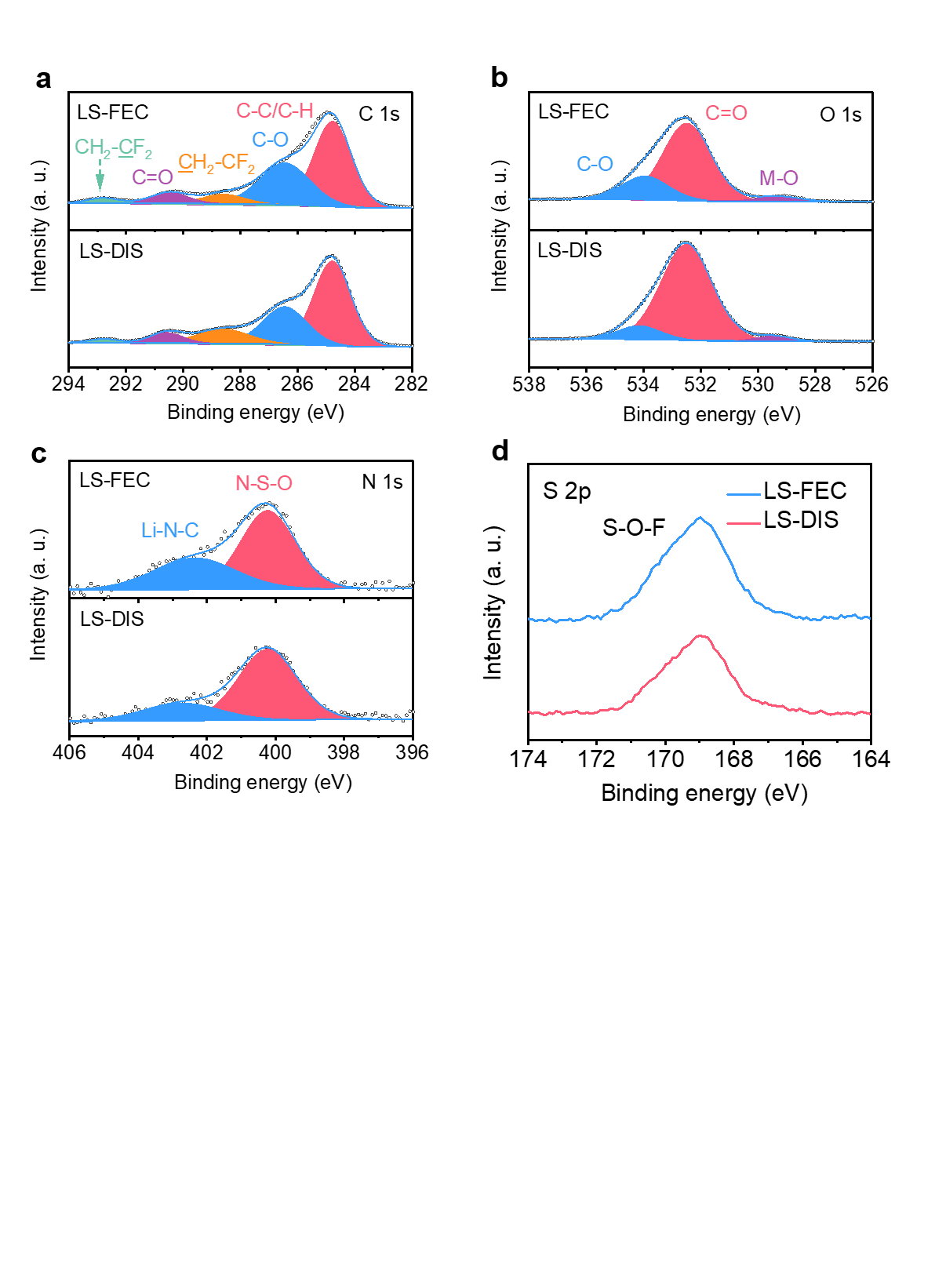


**Figure S29.** (**a**) C 1s, (**b**) O 1s, (**c**) N 1s, and (**d**) S 2p XPS patterns of cycled LCO in LS-FEC and LS-DIS showing the CEI composition.


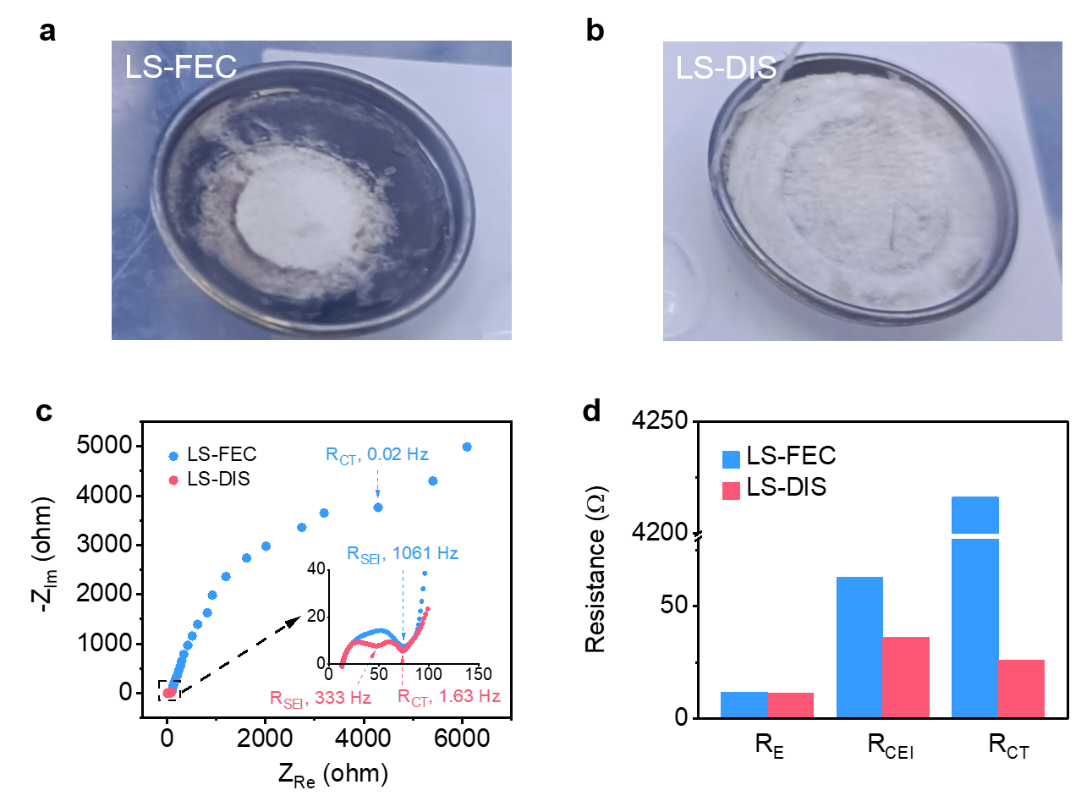


**Figure S30.** Optical images of disassembled cells using the (**a**) LS-FEC and (**b**) LS-DIS. (**c**) EIS patterns and corresponding (**d**) resistance value of cycled LCO in the LS-FEC and LS-DIS.


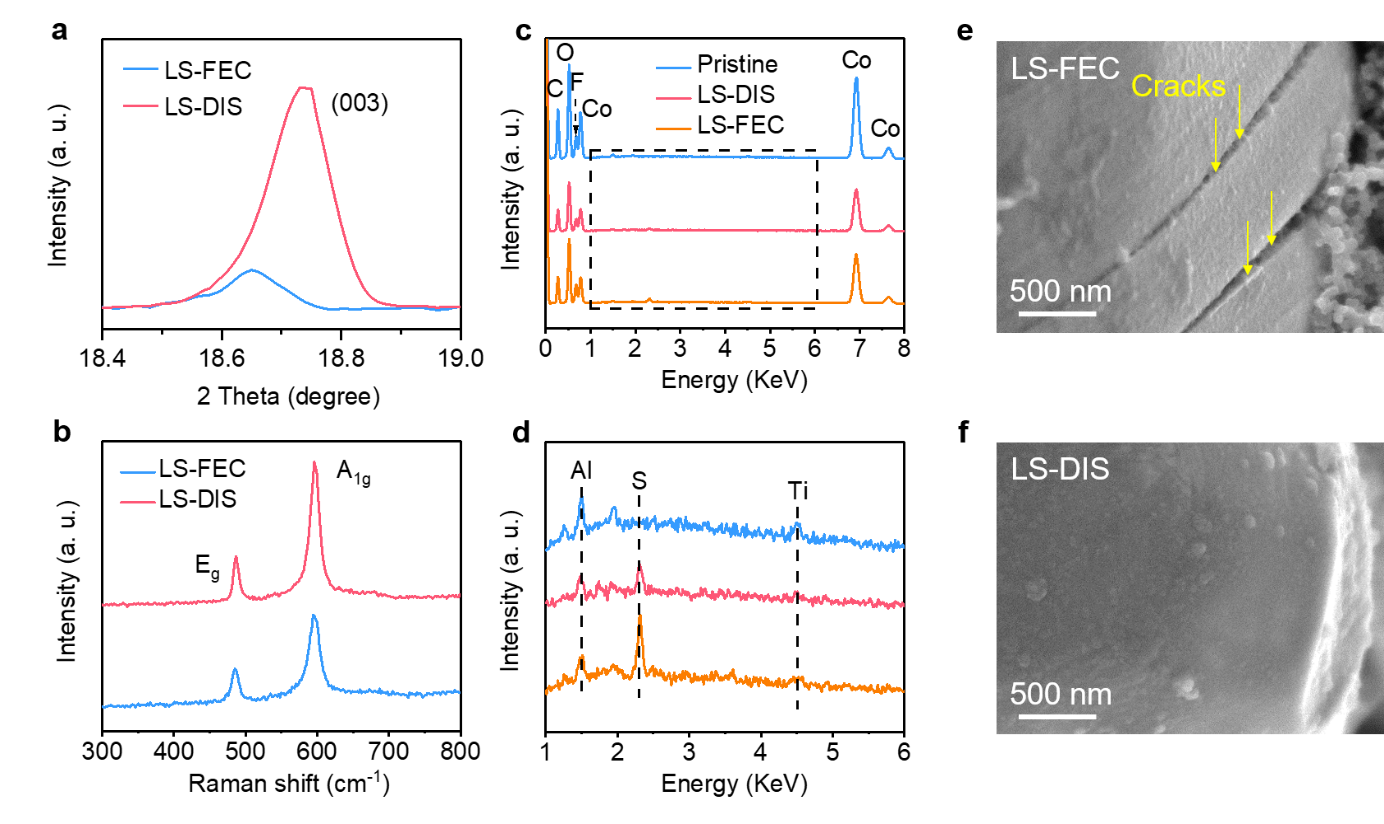


**Figure S31. (a-d**) XRD, Raman, and EDS spectra of LCO after cycling. (**e, f**) SEM images showing the morphology of cycled LCO.


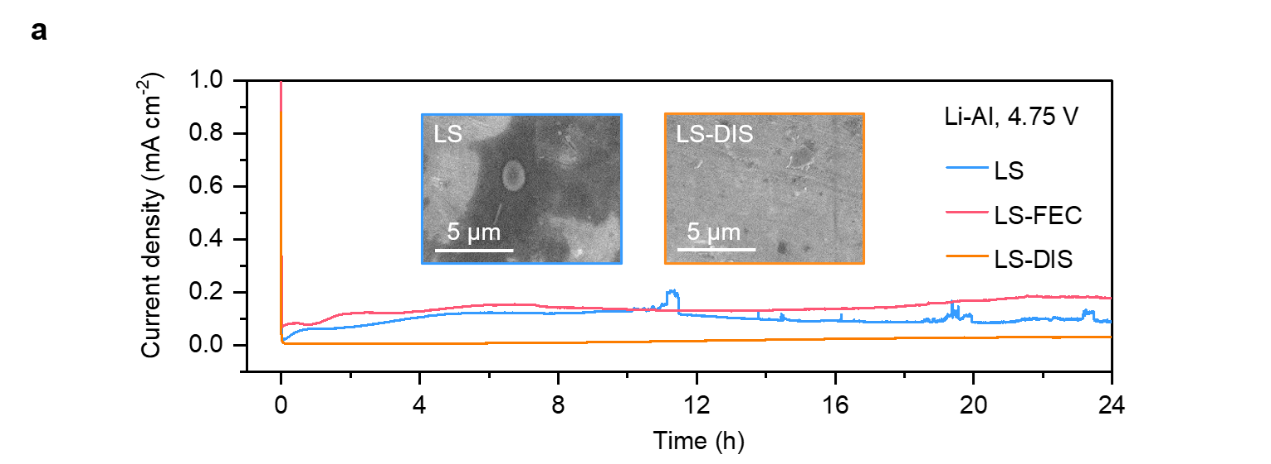


**Figure S32.** Chronoamperometry profiles of Al-Li cells kept at 4.75 V for 24 h. The insets are SEM images of Al foils after the Chronoamperometry test.


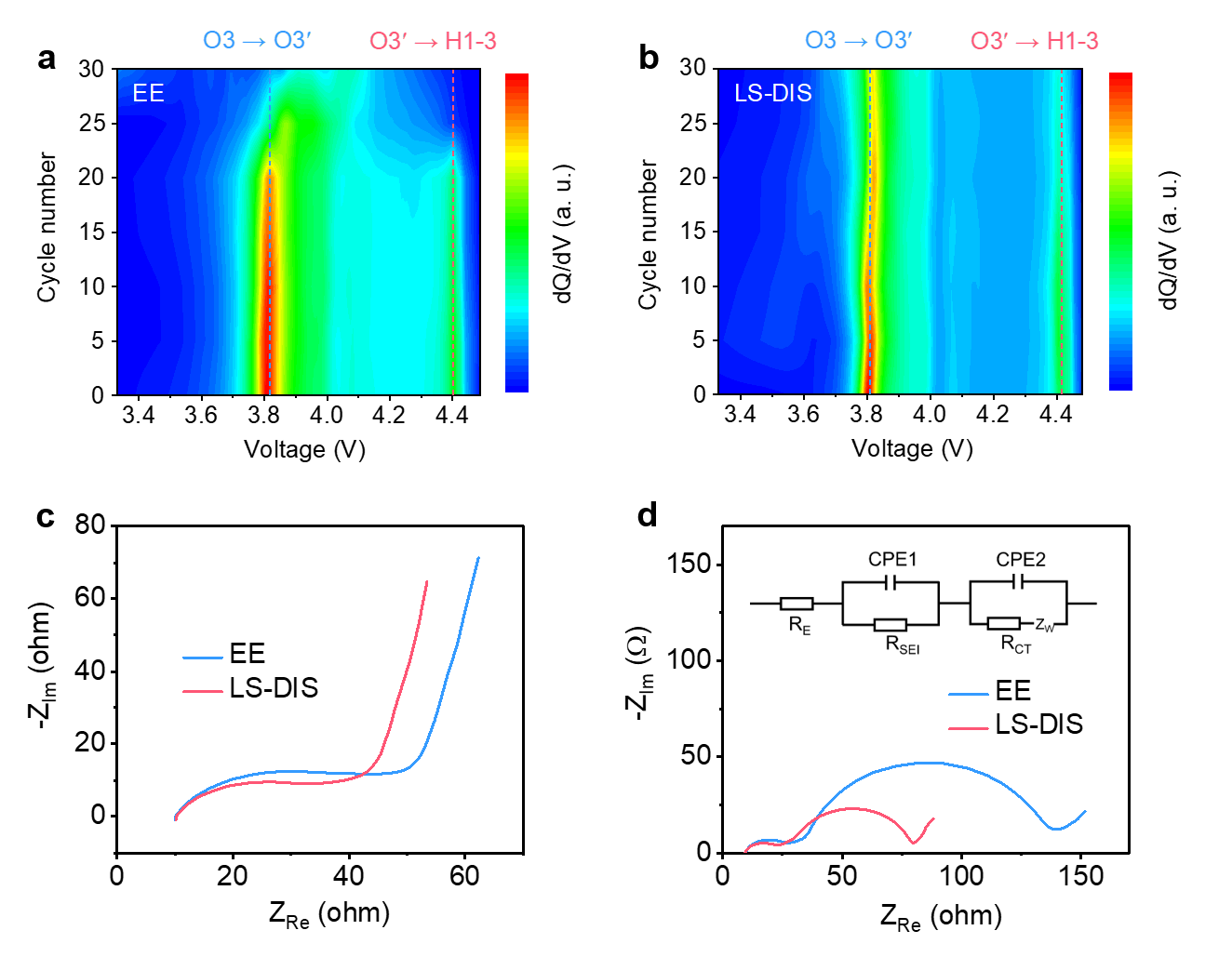


**Figure S33.** (**a, b**) Electrochemical differential plots derived from charge-discharge curves during the initial 30 cycles showing the phase transition of LCO. (**c, d**) Nyquist patterns of pouch cells in the initial state (**c**) and after 30 cycles (**d**). The insets in (**d**) show the equivalent circuit for simulation.


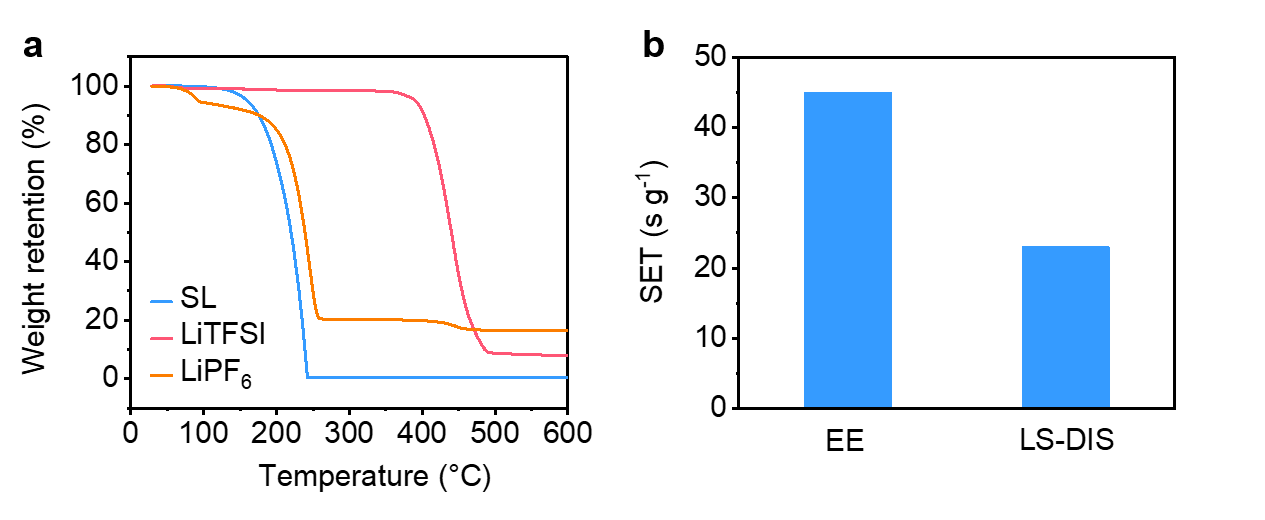


**Figure S34.** (**a**) TGA curves of the SL, LiTFSI, and LiPF_6_ showing the decomposition temperature of these compounds. (**b**) The self-extinguish time (SET) of the EE and LS-DIS.


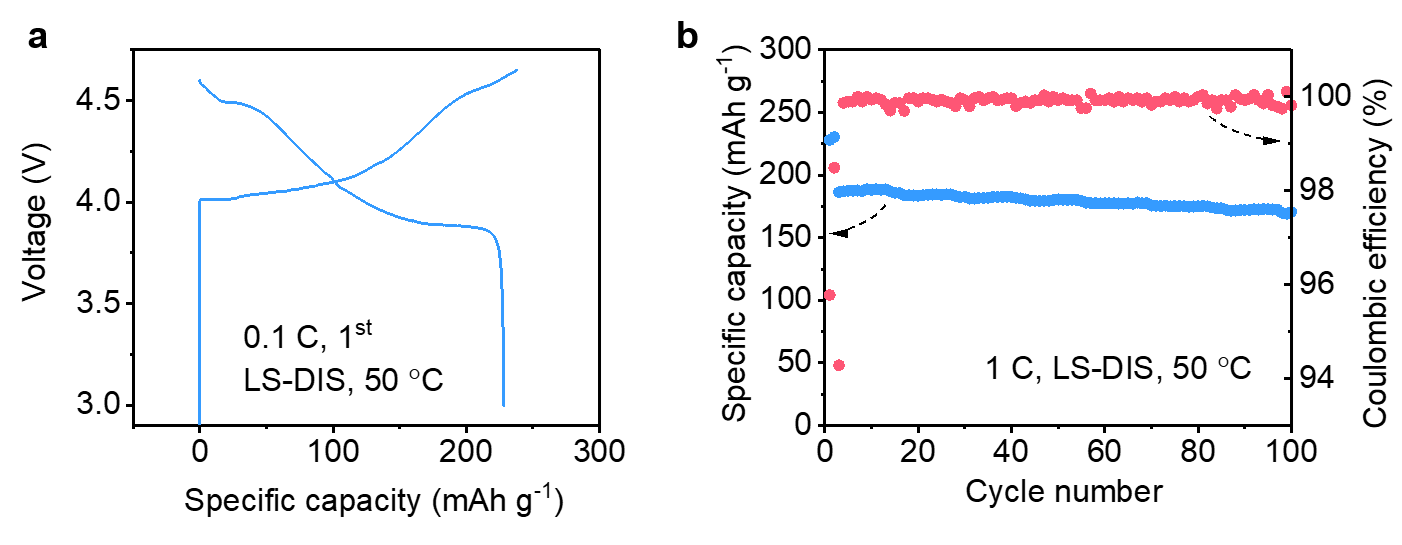


**Figure S35.** (**a-b**) Charge-discharge curves and corresponding cycling performance of high-voltage LCO in the LS-DIS at 50 ℃.

**Table S1.** The theoretical energy density of LCO based on various cut-off voltages.

| **Cut-off voltage**  **(V)** | 4.45 | 4.65 | 4.7 | 4.75 | 4.85 |
| --- | --- | --- | --- | --- | --- |
| **Specific capacity**  **(mAh g^-1^)** | 183 | 239 | 249 | 253 | 257 |
| **Energy density**  **(Wh kg^-1^)** | 666 | 996 | 1064 | 1073 | 1089 |

**Table S2.** The lowest unoccupied molecular orbital (LUMO) and highest occupied molecular orbital (HOMO) energy values from density functional theory calculations.

|  | **LUMO (eV)** | **HOMO (eV)** | **Energy gap (eV)** |
| --- | --- | --- | --- |
| **LiTFSI** | 3.86 | -4.98 | 8.84 |
| **SL** | 1.45 | -9.46 | 10.70 |
| **LiDFOB** | 3.79 | -5.23 | 9.03 |
| **FEC** | 1.24 | -10.87 | 12.32 |

**Table S3.** Li^+^ conductivity and activation energy of various electrolytes at room temperature.

|  | **R (Ω)** | **δ (mS cm^-1^)** | **E_a_ (kJ mol^-1^)** |
| --- | --- | --- | --- |
| **LS** | 8.3 | 1.6 | 21.5 |
| **LS-FEC** | 5.0 | 2.6 | 14.5 |
| **LS-DIS** | 5.8 | 2.3 | 13.1 |

**Table S4.** Price comparisons of SL and common carbonate esters.

| **Solvents** | **Sigma Aldrich** | **Online price**  **(US$)** | **Normalized price (US$/kg)** | **Boiling point (℃)** | **Flash point (℃)** |
| --- | --- | --- | --- | --- | --- |
| **SL** | T22209 | 33.71/100 g | 337.1 | 285 | 165 |
| **EC** | 809950 | 1068.39/500 g | 2136.78 | 243 | 150 |
| **DEC** | 900018 | 1068.39/500 g | 2136.78 | 128 | 33 |
| **DMC** | 809942 | 1068.39/500 g | 2136.78 | 90 | / |
| **EMC** | 809934 | 1068.39/500 g | 2136.78 | 107 | / |

The material prices are referenced from Sigma Aldrich, while boiling points and flash points are referenced from Wikipedia.

**Table S5.** Cycling performance of typical high-voltage LCO with various modification strategies^[5-13]^.

| **Type** | **Material** | **Voltage**  **(V)** | **Cycling retention**  **(%)** | **Decay per cycle**  **(%)** | **Ref.** |
| --- | --- | --- | --- | --- | --- |
| **Doping** | Mg | 4.6 | 84  (100 cycles at 1 C) | 0.16 | [5] |
|  | Al, Ti | 4.6 | 78  (200 cycles at 0.5 C) | 0.11 | [6] |
|  | Mg, Al, Ti | 4.6 | 86  (100 cycles at 0.5 C) | 0.14 | [7] |
| **Coating** | LATP ^a)^ | 4.6 | 88.3  (100 cycles at 0.5 C) | 0.117 | [8] |
|  | DSL ^b)^ | 4.6 | 93.4  (100 cycles at 0.5 C) | 0.066 | [9] |
|  | LAF ^c)^ | 4.6 | 81.8  (200 cycles at 0.1 C) | 0.091 | [10] |
| **Electrolyte** | HTCN ^d)^ | 4.6 | 72  (300 cycles at 1 C) | 0.0933 | [11] |
|  | D-DES ^e)^ | 4.7 | 70  (500 cycles at 1 C) | 0.09 | [12] |
|  | SPFT ^f)^ | 4.65 | 70.3  (300 cycles at 1 C) | 0.099 | [13] |
|  | LS-DIS | 4.65 | 80.8  (500 cycles at 1 C) | 0.0384 | Our work |
|  | LS-DIS | 4.7 | 80.2  (250 cycles at 1 C) | 0.0792 | Our work |
|  | LS-DIS | 4.75 | 80.3  (200 cycles at 1 C) | 0.0985 | Our work |

^a)^ “LATP” represents “Li_1.5_Al_0.5_Ti_1.5_(PO_4_)_3_”; ^b)^ “DSL” represents “dextran sulfate lithium”; ^c)^ “LAF” represents “Li, Al, F-modification”; ^d)^ “HTCN” represents “1,3,6-hexanetricarbonitrile”; ^e)^ “D-DES” represents “dual-anion deep eutectic solution”; ^f)^ “SPFT” represents “potassium (4-methylsulfonylphenyl)trifluoroborate”.

**Table S6.** Cell parameters of the Li-LCO pouch cell.

|  | **Parameter** | **Value** |
| --- | --- | --- |
| **LCO cathode** | Discharge capacity | 228 mAh g^-1^ |
|  | Active material loading | 96.4% |
|  | Area weight | 18.4 mg cm^-2^ |
|  | Area capacity | 4.2 mAh cm^-2^ |
| **Al foil** | Specific mass | 4.2 mg cm^-2^ |
| **Li anode** | Specific capacity | 3,860 mAh g^-1^ |
|  | Li thickness | 50 μm |
|  | Area capacity | 9.8 mAh cm^-2^ |
|  | N/P ratio | 2.3 |
|  | Specific mass | 2.67 mg cm^-2^ |
| **Electrolyte** | E/C ratio | 3 g Ah^-1^ |
| **Separator** | Specific mass | 1.4 mg cm^-2^ |
| **Cell** | Average voltage | 4.1 V |
|  | Capacity | 101 mAh |
|  | Cell energy | 435 Wh kg^-1^ |

**References**

[1] Y. Zhao, D. G. Truhlar, *Theor. Chem. Acc.* **2007**, *120*, 215.

[2] F. Weigend, R. Ahlrichs, *Phys. Chem. Chem. Phys.* **2005**, *7*, 3297.

[3] F. Weigend, F. Furche, R. Ahlrichs, *J. Chem. Phys.* **2003**, *119*, 12753.

[4] S. Grimme, J. Antony, S. Ehrlich, H. Krieg, *J. Chem. Phys.* **2010**, *132*, 154104.

[5] Y. Huang, Y. Zhu, H. Fu, M. Ou, C. Hu, S. Yu, Z. Hu, C. T. Chen, G. Jiang, H. Gu, H. Lin, W. Luo, Y. Huang, *Angew. Chem. Int. Ed.* **2021**, *60*, 4682.

[6] L. Wang, J. Ma, C. Wang, X. Yu, R. Liu, F. Jiang, X. Sun, A. Du, X. Zhou, G. Cui, *Adv. Sci.* **2019**, *6*, 1900355.

[7] J.-N. Zhang, Q. Li, C. Ouyang, X. Yu, M. Ge, X. Huang, E. Hu, C. Ma, S. Li, R. Xiao, W. Yang, Y. Chu, Y. Liu, H. Yu, X.-Q. Yang, X. Huang, L. Chen, H. Li, *Nat. Energy* **2019**, *4*, 594.

[8] Y. Wang, Q. Zhang, Z. C. Xue, L. Yang, J. Wang, F. Meng, Q. Li, H. Pan, J. N. Zhang, Z. Jiang, W. Yang, X. Yu, L. Gu, H. Li, *Adv. Energy Mater.* **2020**, *10*, 2001413.

[9] H. Huang, Z. Li, S. Gu, J. Bian, Y. Li, J. Chen, K. Liao, Q. Gan, Y. Wang, S. Wu, Z. Wang, W. Luo, R. Hao, Z. Wang, G. Wang, Z. Lu, *Adv. Energy Mater.* **2021**, *11*, 2101864.

[10] J. Qian, L. Liu, J. Yang, S. Li, X. Wang, H. L. Zhuang, Y. Lu, *Nat. Commun.* **2018**, *9*, 4918.

[11] X. Yang, M. Lin, G. Zheng, J. Wu, X. Wang, F. Ren, W. Zhang, Y. Liao, W. Zhao, Z. Zhang, N. Xu, W. Yang, Y. Yang, *Adv. Funct. Mater.* **2020**, *30*, 2004664.

[12] Z. Hu, F. Xian, Z. Guo, C. Lu, X. Du, X. Cheng, S. Zhang, S. Dong, G. Cui, L. Chen, *Chem. Mater.* **2020**, *32*, 3405.

[13] Y. Yan, S. Weng, A. Fu, H. Zhang, J. Chen, Q. Zheng, B. Zhang, S. Zhou, H. Yan, C.-W. Wang, Y. Tang, H. Luo, B.-W. Mao, J. Zheng, X. Wang, Y. Qiao, Y. Yang, S.-G. Sun, *ACS Energy Lett.* **2022**, *7*, 2677.
